# Revisiting the structures and phase transitions of Ba_2_NaNb_5_O_15_

**DOI:** 10.1107/S1600576723006969

**Published:** 2023-09-05

**Authors:** Ola G. Grendal, Donald M. Evans, Solveig S. Aamlid

**Affiliations:** a European Synchrotron Radiation Facility (ESRF), 71 avenue des Martyrs, Grenoble, 38000, France; b Experimental Physics 5, Center for Electronic Correlations and Magnetism, Institute of Physics, University of Augsburg, Augsburg, 86159, Germany; c Stewart Blusson Quantum Matter Institute, University of British Columbia, Vancouver, 2355, Canada; HPSTAR and Harbin Institute of Technology, People’s Republic of China

**Keywords:** Ba_2_NaNb_5_O_15_, powder diffraction, density functional theory, DFT, structure determination

## Abstract

The room-temperature and low-temperature structure(s) of Ba_2_NaNb_5_O_15_ (BNN) have been debated since the structure was proposed in the 1960s. This work revisits the structures and phase transitions of BNN, combining high-resolution X-ray and neutron powder diffraction with density functional theory calculations. Temperature-dependent high-resolution X-ray powder diffraction patterns are collected from 4 to 918 K, and sequential batch Rietveld refinement using a symmetry mode approach to describe the structure is used to extract the main structural changes as a function of temperature. The data show that the average structure of BNN is best described by the *Ama*2 space group, and no other structural phase transitions were observed below the ferroelastic transition. The symmetry mode analysis, combining results from diffraction and density functional theory, shows significant octahedral tilting and corrugations of both the *A*1 and *A*2 sites along the **c** direction. A strong correlation between the spontaneous strain and the octahedral tilting was observed, and a potential connection with emerging microstructure at low temperatures is proposed, all enabled by the symmetry mode approach used in this work.

## Abstract 2

Structural changes and phase transitions of ferroelectric Ba_2_NaNb_5_O_15_ are studied using high-resolution X-ray powder diffraction combined with neutron powder diffraction and density functional theory calculations.

## 1. Introduction

Materials with nonlinear properties, such as ferroics (Eerenstein *et al.*, 2006 ▸; Evans *et al.*, 2020 ▸; Salje & Carpenter, 2011 ▸), are of interest for potential device applications. There is particular interest in studying materials whose properties cannot be trivially understood, as this can lead to new physics or emergent phenomena (Weber *et al.*, 2022 ▸). One such material is Ba_2_NaNb_5_O_15_ (BNN) (Jamieson *et al.*, 1969 ▸), for which there is no general agreement on its room-temperature structure, and reports on incommensurate modulations (Schneck *et al.*, 1981 ▸), glassy microstructure (Mori *et al.*, 1997 ▸) and memory effects (Mori *et al.*, 1995 ▸; Manolikas *et al.*, 1987 ▸) further complicate our understanding of the structure–property relations of this compound.

**Figure 1:**
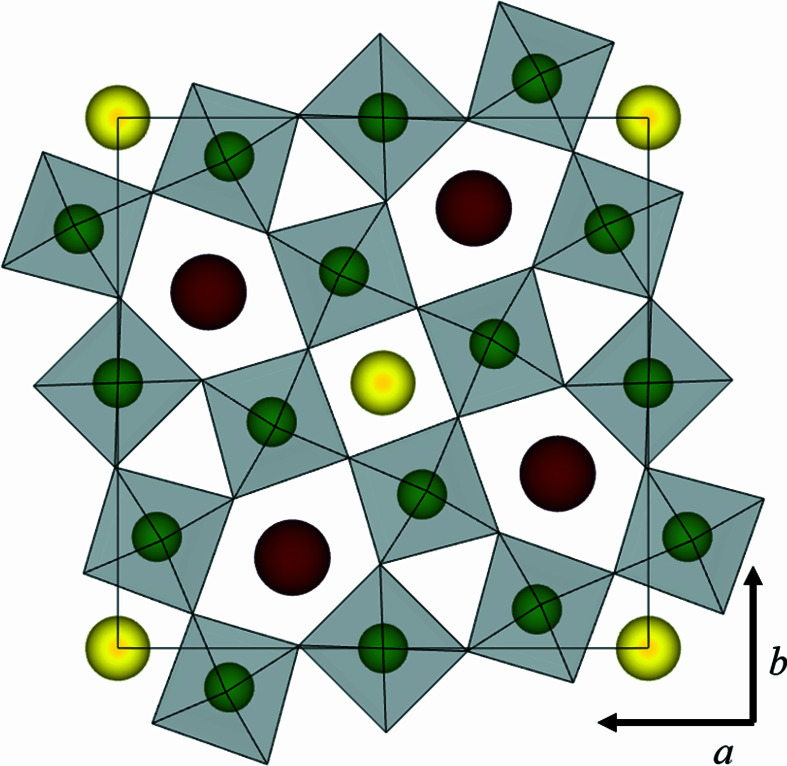
The tetragonal tungsten bronze aristotype non-polar *P*4/*mbm* unit cell. BO_6_ octahedra in grey with B cations at the centre in green (oxygen omitted for clarity), the smaller *A*1 site in yellow and the larger *A*2 site in red. The illustration was made using *VESTA* (Momma & Izumi, 2011 ▸).

It is generally agreed that BNN is a tetragonal tungsten bronze (TTB). The structure of the TTB class of materials is comparable to that of the well known perovskite structure with corner-sharing octahedra but is more complex, with the general formula *A*2_2_ *A*1*CB*1*B*2_4_O_15_. TTBs have attracted increased interest in the search for novel functional materials because their flexible chemistry allows easy tuning, making them an ideal candidate for, *e.g.*, the development of multiferroic and magnetoelectric materials (Rotaru *et al.*, 2014 ▸; Zhu *et al.*, 2015 ▸). BNN is what is known as a ‘filled’ TTB (*i.e.* the *A*1 and *A*2 sites are fully occupied by Na and Ba, while the *C* site is empty), taking on the aristotype non-polar space group *P*4/*mbm* (No. 127) above 840 K (∼570°C) (Jamieson *et al.*, 1969 ▸) (see Fig. 1 ▸). Below 840 K, BNN becomes ferroelectric with the polar space group *P*4*bm* (No. 100), and at around 540 K (∼270°C), the material undergoes a ferroelastic transition to an orthorhombic space group.

While these high-temperature structures of BNN are agreed on, the room-temperature ferroelastic structure has been a source of debate for around 50 years. In the early work by Jamieson *et al.* (1969 ▸) the orthorhombic space group *Cmm*2 (No. 35, with a 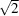 × 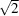 × 1 unit cell relative to the aristotype cell) was suggested, with the added note that it was necessary to include a few split oxygen positions in the refinements. This was explained by potential shearing of the NbO_6_ octahedra along the **c** direction of the unit cell. Later, it was considered that this splitting in oxygen positions was more likely to be from a tilting of the NbO_6_ octahedra along the **c** direction, and that the correct structure should have a doubling of the unit cell along **c**, such as space group *Ccm*2_1_ (No. 36, with a 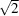 × 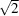 × 2 unit cell relative to the aristotype cell) (Toledano, 1975 ▸). Since then, several space groups have been suggested, all of which are well summarized in the recent work by Whittle *et al.* (2021 ▸). Here we only mention that recent X-ray powder diffraction (XRD) data suggest *Cmm*2 (Aamlid *et al.*, 2020 ▸), while group theory considerations and a thorough review of existing literature suggest *Bbm*2 (No. 40, with a 2 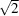 × 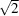 × 2 unit cell relative to the aristotype cell) to be the correct space group (Whittle *et al.*, 2015 ▸, 2021 ▸). The *Bbm*2 space group is also reported experimentally as a good approximation to a true incommensurate structure (Xiao-Qing *et al.*, 1985 ▸; Labbe *et al.*, 1990 ▸). Note that *Bbm*2 is an alternative setting of space group *Ama*2 for which the cell would be 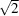 × 2 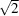 × 2, and in this work, we will refer to the standard setting (*Ama*2) instead of the alternative setting (*Bbm*2) as this simplifies the interface with some of the tools used for the data analysis.

Incommensurate modulations are a common feature for the TTB class of materials (Zhu *et al.*, 2015 ▸; Schneck *et al.*, 1981 ▸), and this is also believed to be a possibility for BNN. Several studies have reported on the existence of satellite reflections below the ferroelastic transition of BNN, consistent with incommensurate modulations of the structure (Schneck & Denoyer, 1981 ▸; Barre *et al.*, 1988 ▸; Kiat *et al.*, 1992 ▸). To the present authors’ knowledge, no structure solution of the incommensurate structure(s) of BNN exists. However, incommensurate structures have been solved and reported for other TTBs (Graetsch *et al.*, 2014 ▸; Graetsch, 2017 ▸). The most noteworthy modulations of these other TTBs were observed for the O atoms and can be described as a cooperative tilting of the NbO_6_ octahedra and cation corrugations on the *A*2 site, both along the **c** direction of the unit cell.

Upon further cooling below room temperature, the challenges in determining the structure(s) of BNN become more complex, with several incommensurate phase transitions reported (Scott *et al.*, 1990 ▸), a reentrant phase transition back to tetragonal (Schneck & Paquet, 1978 ▸; Scott *et al.*, 1990 ▸) and contradictory reports on the existence of these transitions (Filipic *et al.*, 2007 ▸; Fujishiro & Uesu, 1996 ▸). The reentrant tetragonal ‘lock-in’ structure was reported below ∼20 K and described by space group *P*4*nc* (space group No. 104 with a 2 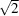 × 2 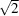 × 2 unit cell relative to the aristotype cell) (Scott *et al.*, 1990 ▸). The discovery that cooling rate had a large effect on the observed (or not observed) phase transitions (Filipic *et al.*, 2007 ▸) led to a discussion of whether it was a true change in thermodynamic state (*i.e.* a phase transition) or an effect of ordering of microstructures whose size and shape would naturally depend on cooling rates. In either case, it is unclear what the physical origin of the low-temperature phase is and why its observation depends so much on the experimental probe used.

In this work we intend to address some of these long-standing issues regarding the room-temperature and low-temperature structure(s) of BNN. We collect high-resolution XRD data to study the structure and phase transitions of BNN over a wide temperature range (4–918 K). Because of the expected (in)commensurate tilting patterns that are typically associated with the oxygen positions, we combine the XRD data with neutron powder diffraction (ND) at two selected temperatures (25 K and room temperature) to increase the sensitivity to the oxygen positions. These powder diffraction data sets are analysed by a symmetry mode approach and combined with density functional theory (DFT) calculations with the aim of settling the debate about the average ortho­rhombic structure of BNN and thus providing accurate structural information, important for further investigations of this intriguing material. This symmetry mode approach also offers unique information about the more minute structural changes as a function of temperature, not only about the phase transitions, and enables a possible explanation for some of the outstanding questions regarding BNN’s structure–property relations.

## 2. Materials and methods

A BNN pellet was prepared with solid-state synthesis by mixing stochiometric amounts of NaNbO_3_, BaCO_3_ and Nb_2_O_5_ and sintering at 1573 K for 2 h. The synthesis is described in detail elsewhere (Aamlid *et al.*, 2020 ▸). Powders were obtained by gently crushing and grinding the as-obtained pellet.

### 2.1. High-resolution X-ray powder diffraction

High-resolution XRD data were collected in transmission geometry on the high-resolution setup at the ID22 beamline, European Synchrotron Radiation Facility, Grenoble, France (Fitch *et al.*, 2023 ▸). Data were collected at 4 K, 25 K and then at 25 K intervals up to 275 K in a liquid-helium-cooled cryostat (12 data collections in total). In the cryostat, one end of the capillary was kept open to the helium atmosphere, to reduce the effect of beam heating (Fitch, 2019 ▸). A hot-air blower was used to control the temperature at room temperature (293 K), 418 K and then every 50 K up to 918 K (12 data collections in total). Diffraction patterns were also collected on cooling; however, we present here only the heating data, unless highlighting something of special interest in the cooling data. A silicon standard (NIST, 640c) was used to calibrate the wavelength (0.3541 Å, 35 keV) and instrumental contribution to peak broadening. The data were collected up to a *Q* of around 11 Å^−1^.

### 2.2. Neutron powder diffraction

Neutron powder diffraction data were collected at the POWGEN diffractometer (BM11-A beamline) at Oak Ridge Spallation Neutron Source (Oak Ridge National Laboratory, Tennessee, USA) (Huq *et al.*, 2019 ▸) through their mail-in program. Approximately 0.25 g of powder was loaded into a 3 mm diameter cylindrical vanadium container. Data were collected at 25 K and room temperature (295 K) for ∼3.5 h at each temperature, with a centre wavelength of 1.5 Å.

### 2.3. Refinement

Simple Pawley refinements with selected space groups (*Cmm*2, *I*4, *Ama*2, *Ima*2 and *Cmc*2_1_) and a conventional Rietveld refinement with the *Ama*2 structure were performed on the room-temperature data using the *TOPAS-Academic* (Coelho, 2018 ▸) software package (version 7). The *Ama*2 structure of BNN was obtained by transforming the polar *P*4*bm* structure using the *TRANSTRU* tool on the *Bilbao Crystallographic Server* (Aroyo *et al.*, 2011 ▸). Lattice parameters, scale factor, zero error, a low-angle peak asymmetry correction (Simple_Axial_Model), and Lorentzian and Gaussian strain contributions to the peak shape were always refined. The background was composed of straight-line segments connecting ∼60 manually selected background points, with one overall scaling factor for all points being refined. The instrumental contribution to peak shapes was modelled with a Voigt function, for which all parameters were fixed to the values obtained from the silicon standard.

Additionally, five thermal parameters (one for each of the four cation sites in the aristotype *P*4*bm* space group, and one for all oxygen atoms), an *A*1–*A*2 cation interchange parameter and all symmetry-allowed atomic *xyz* coordinates were refined for the Rietveld refinement. To account for the highly asymmetric and *hkl*-dependent peak shape (see Fig. S1), a second phase, with parameters constrained by the primary phase but including an additional *hkl*-dependent exponential contribution to the peak shape (exp_conv_const), was introduced, along with its respective scale factor. exp_conv_const had no 2θ dependence. It was found empirically that refining the exponential coefficients in the [*h*00], [0*k*0] and [00*l*] directions independently, and making the remaining coefficients a linear combination of these {*i.e.* the exponential coefficient in the [0*kl*] direction would be ([0*k*0] + [00*l*])/2}, produced greatly improved fits with a minimum of additional free parameters. A conventional method for dealing with such asymmetric peak shapes, *i.e.* a symmetry-adapted spherical harmonics approach, was tested but resulted in worse modelling of the peak shapes than the simple empirical model we have described here. This gave in total 160 independent parameters.

#### 2.3.1. Symmetry mode analysis and temperature dependence

The temperature-dependent refinements were performed as a sequential batch refinement where the output from the refinement at one temperature was used as input for the next, starting from the lowest temperature. It was confirmed that the same results were obtained by performing the same refinements in the reverse order. For this, an approach of refining symmetry-adapted distortion modes was chosen instead of refining the traditional atomic *xyz* coordinates (Kerman *et al.*, 2012 ▸). The main motivations for this choice were firstly to reduce the total number of parameters (the total number of modes is equivalent to the number of free *xyz* coordinates for a given structure, but often just a small subset of the modes are needed to describe the structure), thus stabilizing the refinements, and secondly to extract more physically meaningful information on the temperature-dependent structural changes in BNN. *ISODISTORT*, part of the *ISOTROPY Software Suite* (Stokes *et al.*, 2022 ▸; Campbell *et al.*, 2006 ▸), was used to generate the atomic *xyz* coordinates as linear functions of the displacive symmetry mode amplitudes starting from the *P*4*bm* structure of BNN, so that the symmetry mode amplitudes could be refined directly in *TOPAS*. For *Ama*2 there are 145 displacive symmetry modes.

The modes to be included in the batch refinement were chosen following the procedure described by Kerman *et al.* (2012 ▸), consisting of an initial global optimization strategy described as ‘repeated local minimization from random starting values’ (RLM), followed by ‘mode inclusion’ and ‘mode exclusion’ runs. The RLM involves refining the magnitude of all the symmetry modes together from random starting values, recording their values, binning the values and plotting them as a histogram. The histograms were based on more than 3600 refinement cycles in *TOPAS*. Important modes are those with a clear bimodal distribution or a distribution shifted away from a value of 0 Å. On this basis, an ‘initial mode set’ can be decided.

This is then followed by a mode exclusion run. This involves taking the initial mode set, removing one mode and refining the remaining modes, with the corresponding *R*_wp_ recorded. This process is repeated for each mode in the initial mode set. Modes that can be excluded without significantly increasing *R*_wp_ are then removed from the mode set, giving an ‘excluded mode set’, *i.e.* a set of important modes after the exclusion run.

The mode inclusion run is similar to the exclusion run, but here each mode *not* part of the excluded mode set is included one by one and refined, with the corresponding *R*_wp_ recorded. If a mode significantly reduces *R*_wp_ that mode is deemed important and added to the mode set. This mode set is called the ‘included mode set’. The mode exclusion and inclusion runs were iterated twice, to reduce potential selection bias. More detailed information about the mode selection can be found in the supporting information (all histograms are plotted in Figs. S2–S4 and representative charts plotting *R*_wp_ for the exclusion and inclusion runs are shown in Figs. S5 and S6, respectively).

The process described was performed including both X-ray and neutron powder diffraction data sets to increase sensitivity towards oxygen modes, and they were weighted so that they contributed equally to the overall *R*_wp_. To account for potentially different dominating modes at room temperature and low temperatures, this mode selection was done for data collected at room temperature (293 K) and at 25 K. The two mode sets were combined, and 4 modes were included to fit the high-temperature data. This yielded a total of 40 modes for the ‘final mode set’, presented in Table S1 in the supporting information. This is a significant reduction from the initial 145 modes for *Ama*2 (found at the end of the supporting information).

This final mode set was then used for all the temperature-dependent Rietveld refinements, and modes were constrained to 0 if they are forbidden by symmetry across any of the phase transitions. This gave in total 55 independent parameters for the temperature-dependent refinements below the ferroelastic transition, 28 below the ferroelectric transition and 24 above the ferroelectric transition.

### 2.4. Computational methods

The 368 atom unit cell of BNN in the *Ama*2 setting was relaxed using the *Vienna Ab Initio Simulation Package* (*VASP*) version 5.4.4 (Kresse & Hafner, 1993 ▸, 1994 ▸; Kresse & Furthmüller, 1996*a* ▸,*b* ▸). The projector augmented wave (PAW) (Blöchl, 1994 ▸; Kresse & Joubert, 1999 ▸) method was used in conjunction with the *VASP*-supplied PBE (Perdew *et al.*, 1996 ▸) PAW potentials and the PBEsol (Perdew *et al.*, 2008 ▸) exchange correlation functional. Ba(5*s*, 5*p*, 6*s*), Na(2*p*, 3*s*), Nb(4*s*, 4*p*, 4*d*, 5*s*) and O(2*s*, 2*p*) were treated as valence electrons. The plane-wave basis-set cut-off energy was set to 650 eV, and a gamma-centred *k*-point mesh of 1 × 1 × 2 was used. The electronic convergence condition was set to 10^−8^ eV and the ionic convergence criterion to the norm of all the forces smaller than 10^−4^ eV. Gaussian smearing with a width of 0.01 eV was used for the partial occupancies. All symmetry-allowed ionic positions and the unit-cell geometry and volume were relaxed. The final structure was decomposed into modes and amplitudes with the mode decomposition tool in *ISODISTORT*, using a *P*4/*mbm* cell (relaxed with the same constraints and cell geometry as the *Ama*2 cell) as the parent structure. The resulting modes were matched to the XRD modes.

## 3. Results

### 3.1. Room-temperature structure

**Figure 2:**
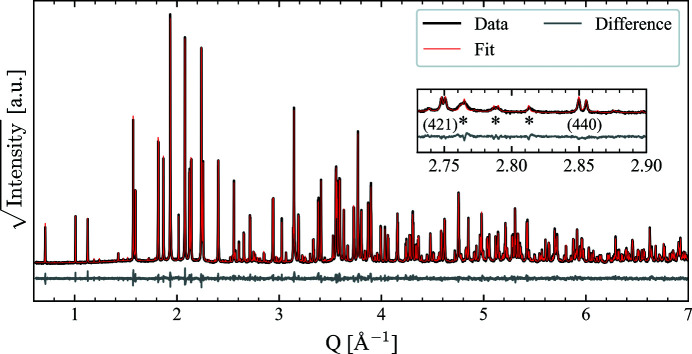
Rietveld refinement of room-temperature X-ray powder diffraction data of BNN using the *Ama*2 structure and refining atomic *xyz* coordinates. The inset shows the pseudo-tetragonal 421 and 440 reflections, with the asterisks indicating some reflections characteristic for *Ama*2 relative to the other space groups investigated. *R*_wp_ = 5.94%, *R*_exp_ = 2.64%, GOF = 2.25. Note that the reflections marked with the two leftmost asterisks are composite reflections.

We start our analysis by determining the room-temperature structure of BNN. On the basis of existing literature, we focused our attention on potential space groups that have their ferroelastic strain along the [110] direction, relative to the parent structure, found to be the most probable according to group theory (Whittle *et al.*, 2021 ▸) and experiments (Aamlid *et al.*, 2020 ▸). An initial Pawley fit screening was performed with the following space groups: *Cmm*2, *I*4, *Ama*2, *Ima*2 and *Cmc*2_1_. All of these space groups described the main features of the diffraction pattern well, with the exception of *I*4, but only *Ama*2 could also fit all the smaller features in the diffraction pattern (see insets in Fig. 2 ▸ and Figs. S10–S14 in the supporting information). *Ama*2 was confirmed as the correct room-temperature space group by performing a Rietveld refinement refining all 145 *xyz* coordinates, the result of which is presented in Fig. 2 ▸. The refinement shows an excellent fit to the data, including the small features highlighted with asterisks in the inset, with the refined lattice parameters being *a* = 17.63794 (2) Å, *b* = 35.20819 (4) Å and *c* = 7.991382 (8) Å. Table S3 in the supporting information contains the refined structural parameters. The refined *A*1 and *A*2 site occupancies are also in good agreement with the literature (Nylund *et al.*, 2022 ▸; Aamlid *et al.*, 2020 ▸), the Ba occupancy on *A*1 being ∼7% and the Na occupancy on *A*2 being ∼4%.

### 3.2. Temperature dependence

**Figure 3:**
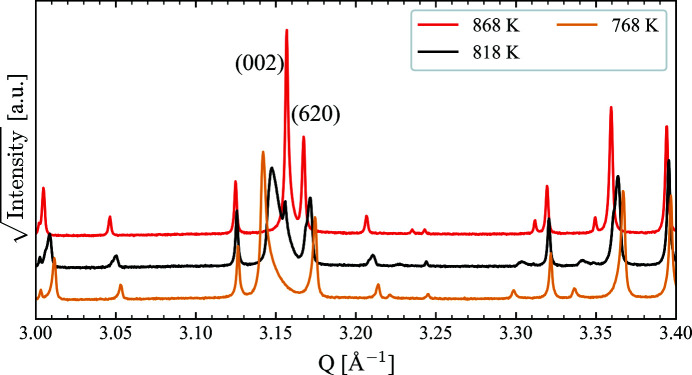
Phase coexistence of the high-temperature *P*4/*mbm* and polar *P*4*bm* structures of BNN upon cooling through the ferroelectric transition. The phase coexistence is most prominent for the 00*l* reflections, since there is a large change in *c* lattice parameter across the ferroelectric transition. This phase coexistence is indicative of a first-order phase transition.

To confirm the high-temperature structures, and to look for the order of the ferroelectric transition, we collected data up to 918 K, on heating and cooling. Above the ferroelastic transition, no deviations from the accepted ferroelectric (*P*4*bm*) and paraelectric (*P*4/*mbm*) structures are observed. Interestingly, while only one phase was present for all temperatures for the heating data, phase coexistence was observed on cooling at 818 K, as shown in Fig. 3 ▸. The transition from *P*4/*mbm* to *P*4*bm* is mainly characterized by a large increase in the *c* lattice parameter, and it is clear that the 818 K data set consists of two phases with significantly different *c* lattice parameters. Indeed, it was confirmed with Rietveld refinement that the 818 K XRD data could be fitted with a combination of the high-temperature *P*4/*mbm* and the polar *P*4*bm* structure of BNN, with differing *c* lattice parameters. Such thermal hysteresis and phase coexistence are generally taken as diagnostic evidence of a discontinuous phase transition and are in agreement with previous work (Aamlid *et al.*, 2020 ▸).

**Figure 4:**
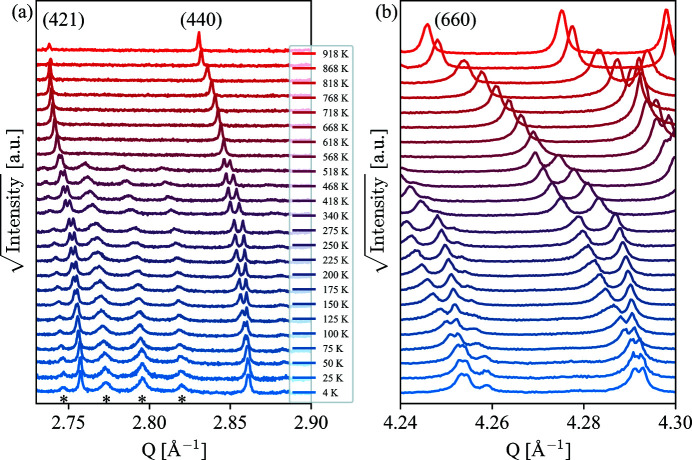
Temperature dependence of the tetragonal (*a*) 421, 440 and (*b*) 660 reflections from X-ray powder diffraction. Clear peak splitting is observed for the three reflections with decreasing temperature, with the 660 reflection staying visibly split down to 4 K. The asterisks in panel (*a*) highlight some of the reflections that are characteristic for *Ama*2 relative to the other structures investigated. Note that the two middle reflections marked with asterisks are composite reflections.

Having established the high-temperature structure (*P*4/*mbm*), the order of the ferroelectric phase transition and the polar structure (*P*4*bm*), we turn our attention to the low-temperature structure(s) of BNN. In Fig. 4 ▸, the temperature evolution of the XRD pattern of BNN is shown for two selected regions. A clear peak splitting and symmetry lowering from tetragonal *P*4*bm* to orthorhombic *Ama*2 is observed for the tetragonal 421, 440 and 660 reflections below 568 K. The magnitude of the orthorhombic distortion increases to a maximum at around 275 K, while below 275 K, the orthorhombic distortion decreases, *i.e.* moves towards a tetragonal-like lattice, but never fully disappears. This is evident from the small, but clear, peak splitting of the 660 reflection remaining at 4 K. At the onset of the peak splitting, the characteristic *Ama*2 reflections are also observed [see asterisks in Fig. 4 ▸(*a*)]. Note that the two middle reflections marked with asterisks are composite reflections. There seems to be a small broadening of these reflections upon cooling relative to the more intense reflections, although the underlying mechanism for this remains unclear. We note that the thermal parameters have a close to linear increase with increasing temperature over the entire temperature range.

**Figure 5:**
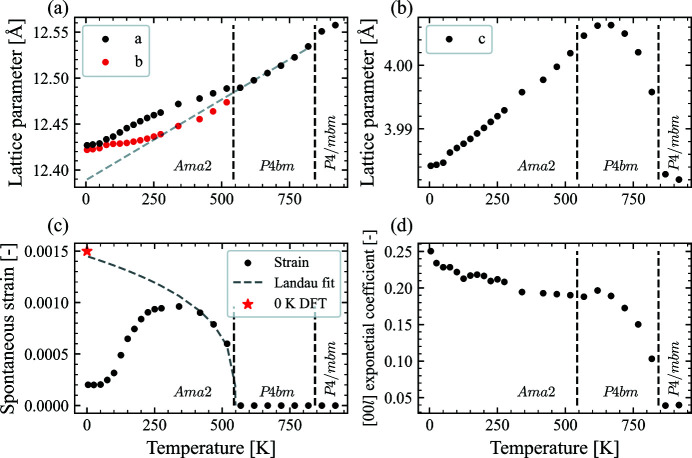
Temperature dependence of (*a*) the *a* and *b* lattice parameters, with a grey dashed line showing the linear fit of the tetragonal *a* lattice parameter over the *P*4*bm* region, extrapolated into the *Ama*2 region, and (*b*) the *c* lattice parameter. Below the ferroelastic transition, the lattice parameters are transposed onto the tetragonal parent structure setting, *i.e. a* = *a*_orth_/ 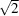 , *b* = *b*_orth_/(2 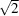 ) and *c* = *c*_orth_/2. (*c*) Spontaneous strain as defined in equation (1), with a grey dashed line from a Landau fit with a critical exponent β of 0.32 (Aamlid *et al.*, 2020 ▸) and a red star marking the strain from the DFT calculation. (*d*) Value of the [00*l*] exponential coefficient accounting for the asymmetric peak shape, demonstrating that the peak shape is a result of the ferroelectric domain structure.

For a more quantitative analysis we use batch Rietveld refinements to look at the temperature dependency of the lattice parameters, spontaneous strain and peak asymmetry. In Figs. 5 ▸(*a*) and 5 ▸(*b*), the temperature-dependent lattice parameters as obtained from the batch Rietveld refinement are presented. The lattice parameters have been transposed onto the tetragonal parent structure setting, *i.e. a* = *a*_orth_/ 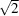 , *b* = *b*_orth_/(2 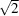 ) and *c* = *c*_orth_ / 2. The *a* and *b* lattice parameters in Fig. 5 ▸(*a*) show the same trend as seen qualitatively in Fig. 4 ▸: an onset of separation below the ferroelastic transition at 518 K, a maximum difference at around 275 K, followed by a decreasing separation which never disappears. The *c* lattice parameter in Fig. 5 ▸(*b*), shows a rapid increase after the ferroelectric transition with a maximum at around 650 K, before following an approximately linear decrease down to the lowest temperatures. A discontinuity can be observed between 50 and 75 K, and a clear change in the slope is observed at this point. A similar discontinuity, although not as apparent, is also observable for the *a* and *b* lattice parameters. A potential origin of this will be discussed later.

The spontaneous strain for the transition from *P*4*bm* to *Ama*2 can be defined as (Carpenter *et al.*, 1998 ▸)

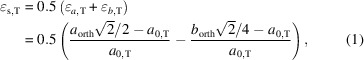

where *a*_orth_ and *b*_orth_ are the *a* and *b* lattice parameters of the orthorhombic phase, while *a*_0,T_ is the extrapolated *a* lattice parameter from the tetragonal phase [plotted as a grey dashed line in Fig. 5 ▸(*a*)]. The spontaneous strain is calculated and shown in Fig. 5 ▸(*c*). The spontaneous strain increases rapidly after the ferroelastic transition, taking on its maximum value at around 275 K and then decreasing again, consistent with the observation of the magnitude of the peak splitting. The spontaneous strain tends towards 0, but the unstrained state is never reached as the strain plateaus at a non-zero value below 75 K. This coincides with the discontinuity in the *c* lattice parameter. As the order parameter of a true ferroelastic phase transition (Salje *et al.*, 2005 ▸), the spontaneous strain, ɛ_s,T_, is proportional to the reduced temperature to the power of the critical exponent, *i.e.* a Landau fit, below the phase transition. This can be defined as

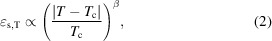

with the critical exponent β = 0.32 for BNN giving an excellent fit in the range between the ferroelastic transition and room temperature (Aamlid *et al.*, 2020 ▸). In equation (2), *T* is the temperature and *T*_c_ is the critical temperature, for which we use 547 K. This relation describes the spontaneous strain well in the range between the ferroelastic transition and room temperature [see the grey line in Fig. 5 ▸(*c*)], but significant deviations are observed below room temperature. Interestingly, the strain predicted from the Landau fit at 0 K is in good agreement with the strain calculated from the DFT relaxed unit cell marked with a red star, while in stark contrast to the experimentally observed spontaneous strain. This could suggest that the driving force for this change in trend is something that is not well captured by DFT, *e.g*. microstructural effects.

The exponential coefficient in the [00*l*] direction, accounting for the asymmetric peak shape, is plotted in Fig. 5 ▸(*d*). This asymmetry is believed to be related to the evolution of ferroelectric domains, as similar effects have been observed for ferroelectric BaTiO_3_ (Floquet *et al.*, 1997 ▸) and various ferroelastic materials (Boysen, 2005 ▸). The coefficient shows a rapid increase after the ferroelectric transition. Closing in on the ferroelastic transition, the rate of change decreases, and below the ferroeleastic transition the value more or less plateaus. Above the ferroelectric transition, the coefficient takes on a value that is essentially 0 (a value of ∼0.02, not noticeable on the peak shape), indicating no peak asymmetry above the ferroelectric transition. This is consistent with visual inspection of the diffraction patterns. Since the temperature-dependent behaviour of the [00*l*] peak asymmetry mimics that of the evolution of the *c* lattice parameter, and the direction is the same as the polarization direction in the uniaxial ferroelectric phase, this asymmetry can be ascribed to the evolution of ferroelectric domains in this material as well.

### 3.3. Symmetry mode analysis based on diffraction and DFT

Although we observe no phase transitions below the ferroelastic transition, we present here the use of symmetry mode analysis to investigate how the *Ama*2 structure of BNN evolves, especially at low temperatures.

**Figure 6:**
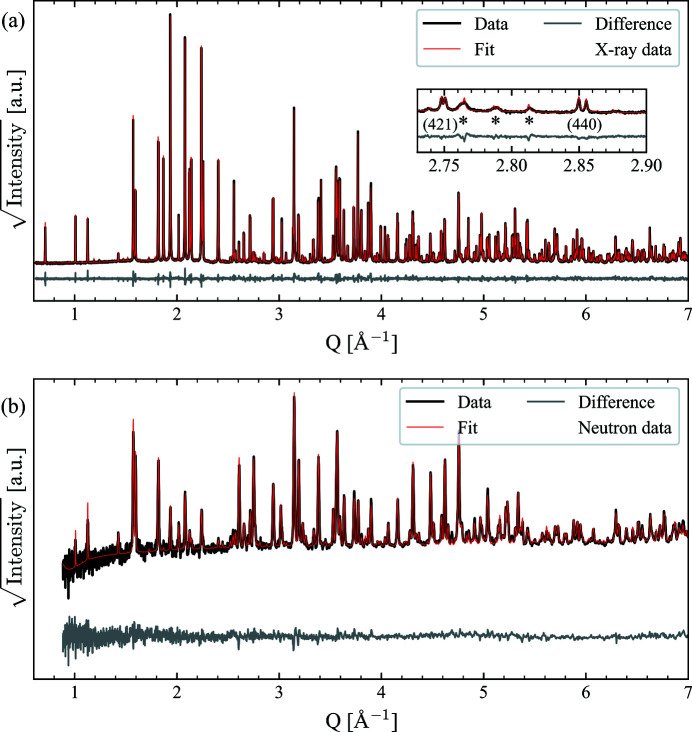
The combined Rietveld refinement of the room-temperature powder (*a*) X-ray diffraction and (*b*) neutron diffraction data refining the 40 symmetry mode amplitudes. *R*_wp_ = 6.09%, *R*_exp_ = 2.04%, GOF = 2.99.

Using the symmetry mode analysis approach described in Section 2.3.1, and detailed in the supporting information, we find that 40 modes are sufficient to describe the BNN structure in the entire temperature range studied in this work. The quality of the fits using this approach is shown for the combined refinement of the room-temperature XRD and ND data in Figs. 6 ▸(*a*) and 6 ▸(*b*), respectively. Even though the number of structural parameters linked to the atomic positions is reduced from 145 to 40 compared with the refinement of the *xyz* coordinates, the fit is excellent, and it is difficult to see any significant differences, both by visual inspection and by comparing the *R* values. This is helpful for our interpretation, as reducing the number of structural parameters should make it easier to pinpoint any temperature-dependent structural changes in BNN.

**Figure 7:**
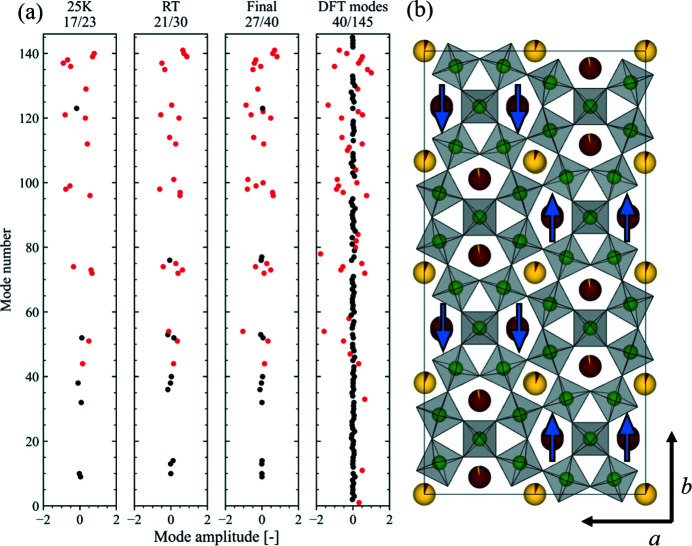
(*a*) Schematic of the diffraction mode selected at 25 K and room temperature and the final mode set, plotted alongside the modes obtained from DFT. The 40 DFT modes with an absolute value greater than 0.13 Å are marked in red, and whenever the same mode is included in any of the diffraction mode sets, it is also marked in red there. Black marks indicate modes that were not selected as one of the 40 DFT modes but are included in the diffraction mode sets. The fractions indicate how many of the selected diffraction modes are also among the 40 DFT modes, *i.e.* number of red modes over number of total modes (red + black). The mode amplitudes are indicated for the DFT modes and the final mode set at room temperature. (*b*) *Ama*2 unit cell with blue arrows indicating the distortion direction of the antiferroelectric mode 44. NbO_6_ octahedra in grey with Nb at the centre in green (oxygen omitted for clarity), Na in yellow (mostly on the square *A*1 site) and Ba in red (mostly on the pentagonal *A*2 site). The illustration was made using *VESTA* (Momma & Izumi, 2011 ▸).

To further emphasize that the selected modes are important in describing the BNN structure, the selected modes are compared with dominating modes in a DFT relaxed unit cell. Assuming that the DFT modes with the largest absolute values are the most ‘important’, we can select the 40 most important DFT modes by selecting those with an absolute amplitude greater than ∼0.13 Å for comparison with the experimentally determined modes. Although the selection criteria are different, *i.e.* based on absolute amplitude and experimental data, it is striking how well the two mode sets match. Out of these 40 modes, 27 (68%) are found in both mode sets. This is visualized in Fig. 7 ▸, where all the DFT modes are presented with the 40 selected on the basis of absolute mode amplitude marked in red, in addition to the modes selected for the 25 K data, the room-temperature data and the final mode set. Note that the good match between diffraction modes and DFT modes is to a large degree because of the excellent match for the oxygen modes (mode numbers 52–145), where 25 out of 30 (83%) diffraction modes match with one of the 34 oxygen DFT modes. Additionally, there is a slightly better match between the low-temperature modes, where 17 out of 23 (74%) match, compared with the room-temperature modes, where 21 out of 30 match (70%) with DFT, as expected since DFT represents a 0 K scenario. Finally, the 40 DFT modes were also used to model the experimental room-temperature XRD data and were found to give a good fit when the mode amplitudes where refined (*R*_wp_ = 6.69%).

**Figure 8:**
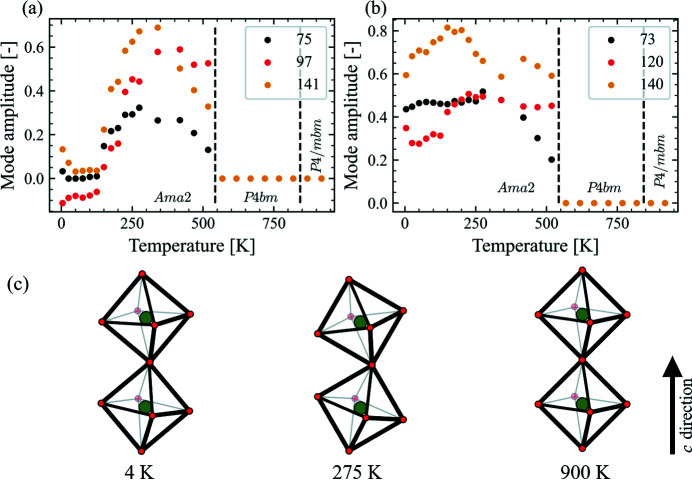
(*a*) and (*b*) Temperature evolution of selected symmetry modes as obtained by temperature-dependent batch Rietveld refinement (all these modes are fixed to 0 above the ferroelastic transition). Modes in (*a*) show a trend similar to the spontaneous strain, while the modes in (*b*) increase rapidly below the ferroelastic transition before plateauing. (*c*) Simplified schematic of the NbO_6_ (O in red and Nb in green) tilting patterns along the **c** direction as a result of the mode amplitudes, illustrating that the degree of tilting goes through a maximum at 275 K coinciding with the maximum in the spontaneous strain. The degree of tilting is exaggerated for visual effect.

Having now confirmed the critical modes for describing the structure, we look at their temperature dependence. Here we focus on a few selected modes that we find particularly interesting, while the description and temperature dependence of all 40 modes are presented in Table S1 and Figs. S15–S17 in the supporting information, respectively. Across the ferroelectric transition, several ferroelectric oxygen modes (modes displacing oxygen atoms along the **c** direction) are activated, associated with irreducible representation (irrep) Γ_1_. Some of these (54 and 101) stay more or less constant down to 4 K after being activated, while others have a slightly increasing amplitude (114 and 124) upon further cooling. In addition to these out-of-plane modes, some modes also associated with irrep Γ_1_ leading to in-plane displacement of oxygen are also activated (52 and 100), showing a temperature dependence reminiscent of that of the *c* lattice parameter. Plotted in Fig. 8 ▸(*a*) are oxygen modes associated with irrep *S*_1_ (75, 97 and 141) that show the same trend as spontaneous strain as a function of temperature, *i.e.* a rapid increase after the ferroelastic transition to a maximum at around 300 K, before decreasing to values close to 0 at 125 K. Interestingly, although they show the same trend as the spontaneous strain, they appear to precede the strain curve, *i.e.* the peak maximum and flattening out at zero occur ∼30 K earlier for these oxygen modes than for the strain. In Fig. 8 ▸(*b*) is a set of modes associated with irrep *S*_1_ (73, 120 and 140) that increase rapidly below the ferroelastic transition before plateauing around 275 K. All six modes can be seen as affecting the NbO_6_ octahedral tilting along the **c** direction of the unit cell. The complete picture of the tilting is somewhat more complex, as more modes are contributing to this effect than the six presented here. A simplified schematic of the combined effect of all modes affecting the octahedral tilting is presented in Fig. 8 ▸(*c*), which provides a good general overview of this complex structure. No tilting modes along the **c** direction are allowed above the ferroelastic transition; *i.e.* for space groups *P*4*bm* and *P*4/*mbm*, a maximum in the tilt is observed around 275 K before the degree of tilting decreases down to 4 K, but remains present.

Another noteworthy observation from the temperature-dependent mode amplitudes is the almost constant value of mode 36 associated with irrep *S*_1_ below the ferroelectric transition, leading to a fixed corrugation of the *A*1 site along the **c** direction of the unit cell. Additionally, modes 38, 40 and 44, associated with irreps Γ_1_, Γ_3_ and *M*_5_, respectively, result in various in-plane shifts of the *A*2 site inside its pentagonal channel. Out of these, mode 44 results in an antipolar ordering [see the blue arrows in Fig. 7 ▸(*b*)], and the possible implications of this mode are described in more detail later. Mode 51, irrep *S*_1_, results in a corrugation of the *A*2 site similar to that produced by mode 36 for the *A*1 site. This mode takes on a constant value below the ferroelastic transition before increasing significantly below ∼275 K all the way down to 4 K, leading to an increasing corrugation of the *A*2 site with decreasing temperature. Although the full picture of the temperature-dependent polarization, octahedral tilting and other structural changes in BNN is still complex, it is encouraging to see how well the symmetry mode approach is able to capture some of the features.

## 4. Discussion

### 4.1. Room-temperature structure

Considering the main question in this work, the room-temperature structure of BNN, the excellent fits to the XRD and ND data obtained by Rietveld refinement make it clear that *Ama*2 is the correct space group below the ferroelastic transition. This is in agreement with recent group theory considerations (Whittle *et al.*, 2021 ▸) and with earlier experimental work (Xiao-Qing *et al.*, 1985 ▸; Labbe *et al.*, 1990 ▸), although it was then only proposed as a good approximation to the true incommensurate structure. Whether *Ama*2 is only a good average approximation of a true incommensurate structure is not easy to determine from powder data, and is also somewhat of a philosophical question that depends on the point of reference.^1^ However, as a simple test to find a potential incommensurate structure matching our data, a Pawley fit with the superspace group *A*2*mm*(½0γ)000 reported for other incommensurate TTBs (Graetsch *et al.*, 2014 ▸; Graetsch, 2017 ▸) was performed using *Jana2020* (Petříček *et al.*, 2014 ▸), and the resulting fit is shown in Fig. S18. This gave inconclusive results, as *A*2*mm*(½0γ)000 did not give fully satisfactory fits to the weaker reflections, even when including second-order satellite reflections. This does not prove that BNN is commensurate or incommensurate, only that the incommensurate structure found to fit other TTBs does not fit for BNN. To summarize, *Ama*2 gives an excellent fit to the data in this work, which give strong evidence that this is the correct average structure of BNN, but it cannot be ruled out that this is no more than a very good approximation to a true incommensurate structure.

### 4.2. Low-temperature reentrant phase transition

In terms of low-temperature phase transitions, the temperature-dependent diffraction data show no phase transitions below the ferroeleastic transition and show clearly that BNN is still orthorhombic at 4 K. Others have noted that the assumed reentrant tetragonal phase only appears under slow cooling conditions (Filipic *et al.*, 2007 ▸). The data presented in this work were all collected during heating, and cooling to 4 K from room temperature was done as quickly as possible, taking approximately ∼1 h. However, the same data collection was also performed during a second cooling cycle, with a total cooling time from room temperature to 4 K of ∼10 h, and the low-temperature data show the same orthorhombic structure when cooled slowly. This is with a slower cooling rate than what was reported to be sufficient to observe the low-temperature tetragonal ‘lock-in’ phase (Filipic *et al.*, 2007 ▸).

### 4.3. Temperature dependence and symmetry mode analysis

Although no evidence for a reentrant phase transition is observed, the data do show noteworthy structural changes with decreasing temperature. For example, below the ferro­elastic transition the *a* and *b* lattice parameters diverge to a maximum separation at ∼275 K. This divergence is then also accompanied by a maximum in the spontaneous strain, consistent with the spontaneous strain being the order parameter of the phase transition. Below this temperature the difference in the *a* and *b* lattice parameters decreases, *i.e.* the orthorhombic distortion decreases, and thus also the spontaneous strain. One might expect an order parameter (such as the spontaneous strain for the tetragonal to orthorhombic phase transition) to continue its trend unless some competing driving force starts dominating. This is what DFT predicts for example, as shown in Fig. 5 ▸(*c*). With this in mind, it is interesting to observe several symmetry modes following a similar trend, *i.e.* an in-plane displacement mode for the *A*2 site (mode 40) and the oxygen modes presented in Fig. 8 ▸(*a*). It is obviously difficult to determine what is the underlying driving force and what is an effect of this driving force, but it seems clear that, although the trends are the same, there is a shift to higher temperatures for the oxygen modes, suggesting this happens first and then the lattice parameters follow. This could make sense as an increasing degree of tilting of the NbO_6_ octahedra along the **c** direction should result in an increase in the *a* and *b* lattice parameters, and thus also more space for an in-plane displacement of the *A*2 site, and *vice versa*. In addition to these tilting modes, several ferroelectric modes are activated below the ferroelectric transition and remain present to the lowest temperatures, further complicating the structural analysis.

It is also interesting that this unusual behaviour of the *a* and *b* lattice parameters and the emergent microstructure are reported to appear in the same temperature interval in BNN (Mori *et al.*, 1997 ▸). This behaviour is not captured by the DFT calculations. We cannot say if this is anything more than a coincidence, but DFT is not sensitive to microstructural effects and diffraction is indirectly sensitive, only if it affects the structure. It seems clear that there are some underlying driving force affecting the structure of BNN that are not captured well by DFT. We suggest, on the basis of an active antipolar mode being found both experimentally and by DFT, *e.g.* mode 44 (irrep *M*_5_) shown schematically in Fig. 7 ▸, that the reported microstructure (Mori *et al.*, 1997 ▸) could be linked to antipolar domains forming inside the ferroeleastic domains. Although such a mode does not give a net change in dipole moment within one unit cell, it does break the degeneracy for the same modes in the surrounding cells, *i.e*. nucleation of antipolar correlations. Such correlations would be separated by antiphase boundaries, like those observed in the literature for BNN (Mori *et al.*, 1997 ▸). Naturally, any ordering of this kind would be within ferroelastic domains, and so any thermal cycling that changed the ferroelastic domain structure would also change this local ordering, giving different macroscopic properties. This would then be consistent with a range of previous observations, like the existence of glass-like microstructure (Mori *et al.*, 1997 ▸), different correlation lengths with different cooling rates (Fujishiro & Uesu, 1996 ▸), specific heat measurements (Filipic *et al.*, 2007 ▸), memory effects (Mori *et al.*, 1995 ▸; Manolikas *et al.*, 1987 ▸) and even changes of in-plane dielectric properties if the microstructure becomes ‘locked-in’ (Filipic *et al.*, 2007 ▸). Furthermore, the discontinuity visible for the *c* lattice parameter between 50 and 75 K, accompanied by anomalies for the *a* and *b* lattice parameters and several of the modes (*e.g.* 75, 97 and 141, shown in Fig. 8 ▸), could be a signature of this glassy microstructure (Filipic *et al.*, 2007 ▸).

Additionally, although we have considered an average structure for BNN, it is encouraging to observe that the main structural motifs, *i.e.* tilting of NbO_6_ octahedra and corrugations of the *A*2 sites along the **c** direction, extracted with the symmetry mode approach are in excellent agreement with the structural motifs reported for incommensurately modulated TTBs (Graetsch *et al.*, 2014 ▸; Graetsch, 2017 ▸) and early suggestions that a doubling of the unit cell relative to the aristotype structure is needed for BNN (Toledano, 1975 ▸). It is also encouraging to see the good agreement between our experimentally determined symmetry modes and those deemed important according to DFT calculations.

Finally, group theory considerations make a strong argument that the most likely tilt systems for TTBs are those where the tilt angles are the same, or nearly the same, for all octahedra (Whittle *et al.*, 2018 ▸, 2021 ▸). The symmetry mode approach used in this work gives experimental evidence in support of this. Although we have just used a subset of all the modes describing the *Ama*2 structure of BNN, the modes selected, their amplitudes and their combined effect on the NbO_6_ octahedra suggest that the tilt angles are nearly the same for all octahedra.

## 5. Conclusions

The structures and phase transitions of BNN have been studied with a combination of temperature-dependent high-resolution XRD and ND measurements, in addition to DFT calculations. The XRD data show that the average room-temperature structure is well defined by the *Ama*2 space group, thus potentially settling a 50-year-old debate. However, it cannot be completely ruled out on the basis of the current work that this is just a good average approximation to a true incommensurate structure.

Symmetry mode analysis was used to study the structural changes as a function of temperature, with the selected modes being in excellent agreement with symmetry modes obtained from DFT and structural motifs for incommensurately modulated TTBs. The structure is observed to have an increasing degree of spontaneous strain and octahedral tilting when cooling below the ferroelastic transition to 275 K, before, somewhat unusually, changing to a decreasing trend below 275 K. Although we do not pinpoint the driving force for such a change, we do hypothesize that the octahedral tilting is leading this change, that the spontaneous strain changes as a result of this, and that there is a strong link between structural changes and microstructural changes in this material. In addition to the octahedral tilting, the symmetry mode analysis shows that both the *A*1 and *A*2 sites have a corrugation along the **c** direction of the unit cell. The symmetry mode analysis also shows an active antiferroelectric mode, which potentially explains some of the questions regarding glassy microstructure, specific heat measurements and memory effects reported for BNN in the literature.

Lastly, the elusive low-temperature reentrant tetragonal ‘lock-in’ phase reported for BNN was not observed, but as mentioned, we do see a marked decrease in the orthorhombic distortion with decreasing temperature. We respectfully propose that the previous observations of this reentrant phase transition could be a result of resolution limits of the data, as the orthorhombic distortions are small at the lowest temperatures for BNN.

## Supplementary Material

Additional details about the asymmetric and hkl-dependent peak shape, the displacive symmetry-mode search, the Pawley fits of selected space groups, the refined parameters for BNN with the Ama2 space group at room temperature, the temperature-dependent symmetry mode amplitudes, the Pawley fit of an incommensurate structure, and the full list of symmetry modes for Ama2. DOI: 10.1107/S1600576723006969/iu5044sup1.pdf

## Footnotes

1
*I.e.* satellite reflections, arising from both commensurate and incommen­surate modulations, is a term that only makes sense in relation to a space group that fails to index these reflections. If a different choice of space group is made that indexes these reflections, they are just reflections as any other, as is the case for all the reflections observed in this work when using the *Ama*2 space group.
