# Supplementary material for: Revisiting the structures and phase transitions of Ba2NaNb5O15
Source: J Appl Crystallogr. 2023 Sep 5;56(Pt 5):1456–65. doi: 10.1107/S1600576723006969 (PMC10543678; doi:10.1107/S1600576723006969)
Supplement: Supplementary file 1 [file j-56-01456-sup1.pdf]

JOURNAL OF
APPLIED
CRYSTALLOGRAPHY

Volume 56 (2023)

Supporting information for article:

Revisiting the structures and phase transitions of $Ba_2NaNb_5O_{15}$

Ola G. Grendal, Donald M. Evans and Solveig S. Aamlid

Asymmetric and *hkl*-dependent peak shape

For all X-ray diffraction patterns collected below the ferroelectric transition, asymmetric and *hkl*-dependent peak shapes were observed. The long peak tails on the high-angle side of the [00*l*] reflections were most pronounced, as shown in Figure S1, although all reflections with a non-zero *l* had varying degree of asymmetry. Similar peak asymmetry has been reported for ferroelectric $BaTiO_3$ [1] and various ferroelastic materials [2] and are believed to arise due to the domain structure. From this, and due to the very clear onset of the peak asymmetry below the ferroelectric transition, it is evident that the peak asymmetry observed for barium sodium niobate ($Ba_2NaNb_5O_{15}$, BNN) is linked to the ferroelectric domain structure. In this work, a purely empirical approach was used to fit the peak shape, using a *hkl*-dependent *exp_conv_const* peak shape convolution. The effect of this approach is highlighted in Figure S1.

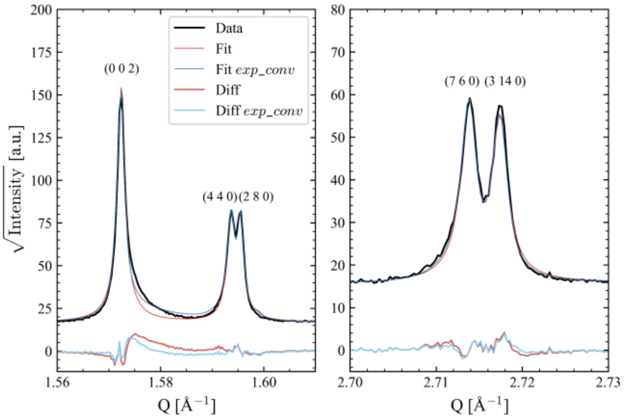

Figure S1. Selected reflections showing the peak asymmetry for the {00l} reflections. The fits obtained by Rietveld refinement using a simple and symmetric peak broadening due to strain are shown in red. The fits including the *exp_conv_const* convolution are shown in blue, greatly improving the peak shape description of the (002) reflection.

Displacive symmetry-mode search

For the temperature dependent batch refinements, it was decided to refine *symmetry-adapted distortion modes* instead of refining the traditional atomic *xyz* coordinates. This was done to reduce the number of parameters in the Rietveld refinement, thus stabilizing the refinements, and to obtain more physically meaningful information on the structural changes in BNN as a function of temperature. For a given structure there is a one-to-one relationship between the number of free atomic *xyz* coordinates, and the number of symmetry-modes. However, it is normally possible to find a significantly smaller subset of the total number of symmetry-modes, that fits the data equally well, thus reducing the number of parameters [3].

In this work we used X-ray and neutron diffraction data at both room temperature and 25 K to find a subset of the symmetry-modes. The X-ray and neutron data were modeled as described in the main text, with the *hkl*-dependent asymmetric peak shape only being applied to the X-ray data and the lattice parameters for the neutron data free, but restrained to the lattice parameters of the X-ray data (to account for slightly different temperatures). The method for selecting symmetry modes follows from the work of Kerman *et al.* [3], and can be divided into three parts: repeated local minimization from random starting values (RLM), exclusion mode run, and inclusion mode run. During the symmetry-mode search, all other parameters than the symmetry-mode amplitudes and scale factors were kept fixed to values obtained from a refinement with all symmetry-modes amplitudes fixed to 0. *ISODISTORT*, part of the *ISOTROPY Software Suite* [4,5], was used to generate the atomic *xyz* coordinates as linear functions of the displacive symmetry-mode amplitudes, so that the symmetry-modes could be refined directly in *TOPAS*. There are in total 145 displacive symmetry modes for *Ama*2. To keep the origin constrained, the three ferroelectric modes acting on the B1-site (GM1, GM3 and S1) were fixed to 0, leaving 142 symmetry modes.

RLM involves refining all symmetry modes together from random starting values, recording all the values upon convergence, and repeating this process a number of times. In the work by Kerman *et al.* [3], ten times the number of free parameters was reported as a good number of cycles to get a good sampling of the modes. In this work, ~3600 cycles were performed, which is approximately 25 times the number of free parameters. The staring values were randomized in the interval –0.1 Å and 0.1 Å. To suppress the inclusion of unimportant modes, a penalty of the form $ln(|mode\_amplitude| + 1)$ was applied to all the symmetry-modes. The multi-convergence mode-amplitude histograms for each symmetry mode is presented in Figures S2-S4, with the symmetry modes selected based on either a bimodal distribution and/or a shift away from 0, for the *initial mode set* being highlighted in green. The initial mode set consisted of the following 57 modes: 11, 14, 22, 27, 30, 31, 32, 33, 36, 37, 38, 39, 40, 44, 47, 51, 52, 53, 54, 55, 72, 73, 74, 75, 76, 78, 81, 88, 90, 92, 96, 97, 98, 99, 101, 104, 106, 107, 110, 112, 114, 115, 116, 117, 120, 121, 122, 124, 125, 126, 127, 135, 136, 137, 139, 140, 141 (see end of this document for full list of all *Ama*2 modes).

The mode exclusion run involves excluding (*i.e.* fixing to 0) one by one the symmetry modes from the refinement and record the R_{wp} values. For each symmetry mode, ten refinements cycles were performed from randomized starting values, and only the lowest R_{wp} value was recorded. The symmetry modes that does not significantly increase the R_{wp} can be removed from the initial mode set. Figure S5 shows the symmetry modes sorted from high to low R_{wp} values for a representative mode exclusion run. Based on this, 29 modes were excluded: 11, 22, 27, 30, 31, 32, 33, 37, 39, 55, 76, 81, 88, 90, 92, 104, 106, 107, 110, 112, 115, 116, 117, 122, 124, 125, 126, 127 and 136. This new mode set is now referred to as the *excluded mode set*.

The mode inclusion run is similar to the mode exclusion run, but here all the modes not part of the excluded mode set are included one by one, and the R_{wp} value recorded. The symmetry modes that significantly reduces the R_{wp} value are included. Figure S6 shows the symmetry modes sorted from low to high R_{wp} values for a representative mode inclusion run. Based on this, 6 modes were included: 10, 13, 31, 76, 124 and134. This new mode set is now referred to as the *included mode set*.

The RLM was only done for the room temperature data, while two cycles of mode exclusion and mode inclusion runs were performed both on the room temperature data and the 25 K data. For the room temperature data 30 modes were selected, while for the 25 K data 23 modes were selected, only

6 of which were not found for the room temperature data. The refinements of the room temperature data and the 25 K data with their respective final mode sets are presented in Figures S8 and S9. Combining these mode sets gave 36 modes, to which 4 modes allowed in the *P*4/*mbm* space group that were not already selected were added, resulting in a *final mode set* consisting of 40 modes. All the selected modes are presented in Table S1.

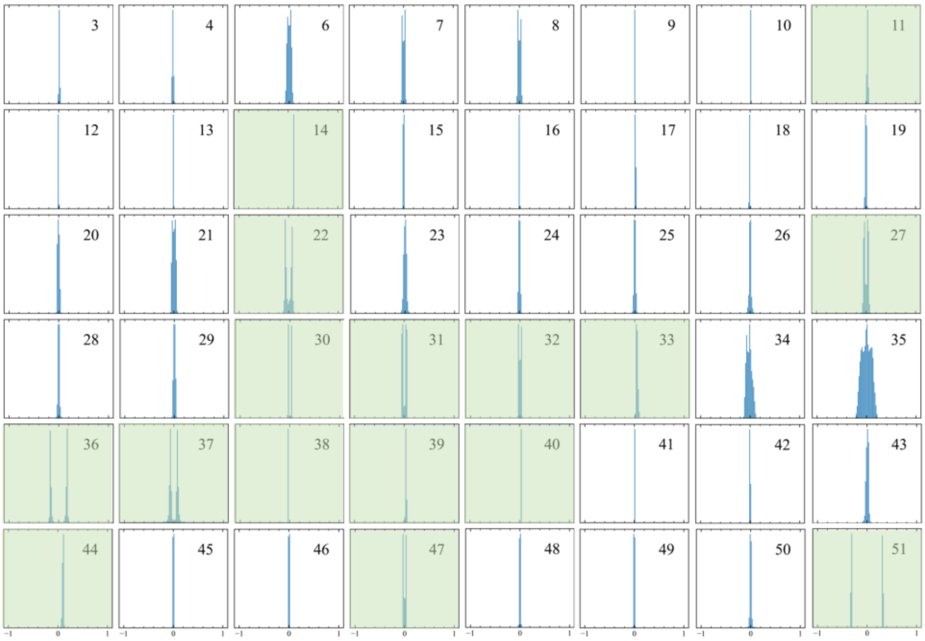

Figure S2. Multi-convergence mode-amplitude histograms for each of the displacive-mode amplitudes for the cations in BNN. The displacive modes highlighted in green was selected to the initial mode set. The horizontal axis of each plot goes from -1 to 1 Å, with tick marks every 0.2 Å, while the vertical axis indicate frequency in arbitrary units. With respect to the polar *P*4*bm* structure of BNN, modes 3-8 acts on B1/b, modes 9-32 on B2/d, modes 33-37 on A1/a and 38-51 on A2/c.

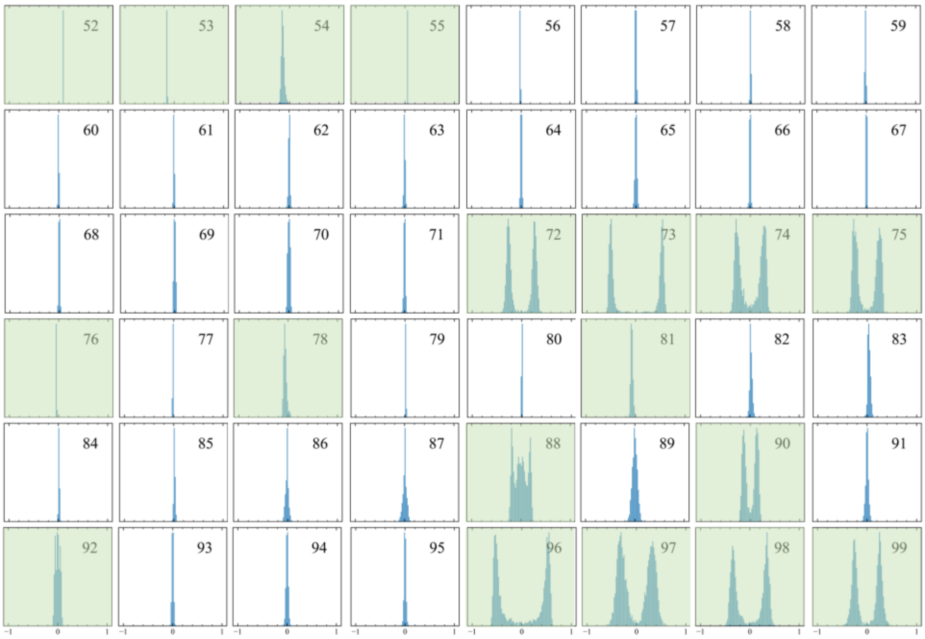

Figure S3. Multi-convergence mode-amplitude histograms for each of the displacive-mode amplitudes for the O1 and O2 oxygen sites in BNN. The displacive modes highlighted in green was selected to the initial mode set. The horizontal axis of each plot goes from -1 to 1 Å, with tick marks every 0.2 Å. With respect to the polar *P*4*bm* structure of BNN, modes 52-75 acts on O1/d, modes 76-99 on O2/d.

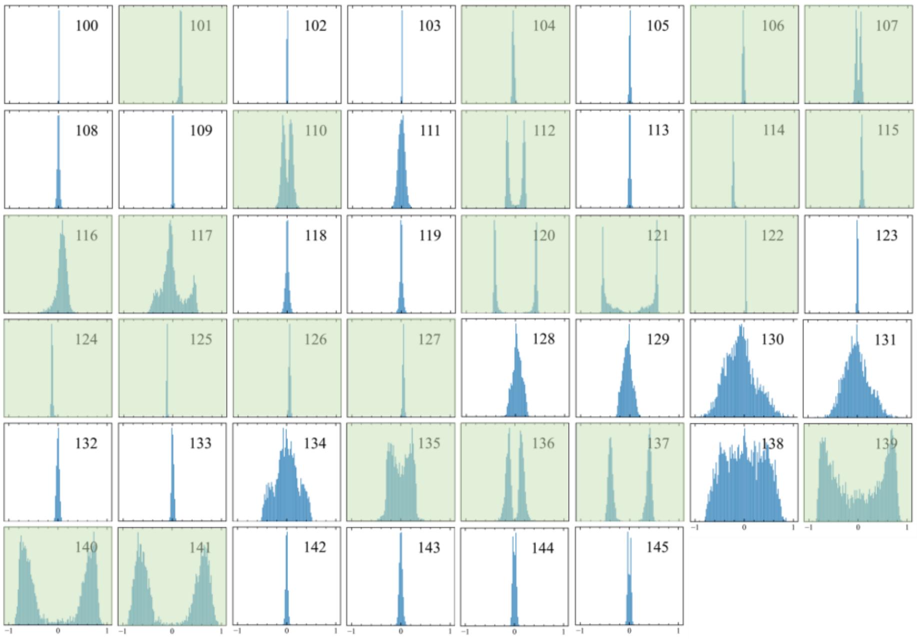

Figure S4. Multi-convergence mode-amplitude histograms for each of the displacive-mode amplitudes for the O3, O4, and O5 oxygen sites in BNN. The displacive modes highlighted in green was selected to the initial mode set. The horizontal axis of each plot goes from -1 to 1 Å, with tick marks every 0.2 Å. With respect to the polar *P*4*bm* structure of BNN, modes 100-113 acts on O3/c, modes 114-121 on O4/b, modes 122-145 on O5/d.

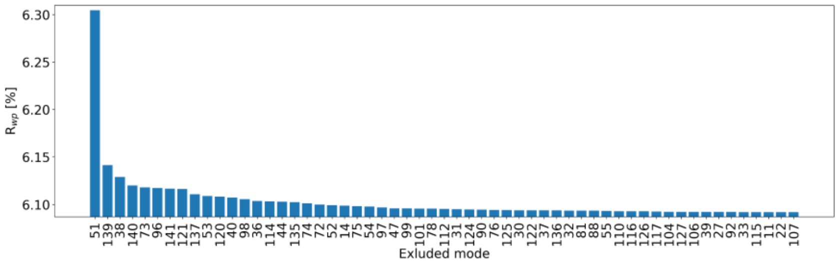

Figure S5. Mode exclusion chart for the initial mode set. Based on this, the modes impacting the R_{wp} value less than mode 78 were excluded, 29 in total.

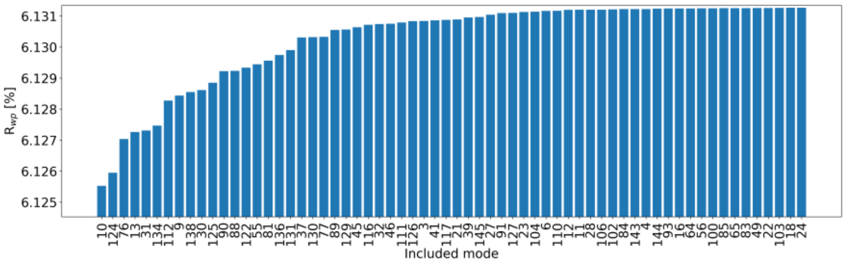

Figure S6. Mode inclusion chart for the initial mode set. Based on this, the first six modes were included, namely 10, 124, 76, 13, 31 and 134. The 50 least significant modes have been omitted from the figure for clarity.

Table S1. The final mode set used for the temperature-dependent batch refinements. Mode number in the *Ama*2 space group, the irreducible representation (IRREP), the components of the order parameter direction vector of the IRREP, resulting space group if this symmetry mode acted alone, the atom acted on and the Wyckoff position of the atom in the parent space group.

Mode # (*Ama*2)	IRREP	IRREP direction	Res space group	Atom	Wyckoff position
9	Γ_1	(a)	*P*4*bm*	Nb2	*d*
10	Γ_1	(a)	*P*4*bm*	Nb2	*d*
13	Γ_3	(a)	*Cmm*2	Nb2	*d*
14	Γ_3	(a)	*Cmm*2	Nb2	*d*
32	S_1	(a,0,0,0)	*Ama*2	Nb2	*d*
36	S_1	(a,0,0,0)	*Ama*2	A1	*a*
38	Γ_1	(a)	*P*4*bm*	A2	*c*
40	Γ_3	(a)	*Cmm*2	A2	*c*
44	M_5	(a,a)	*Pma*2	A2	*c*
51	S_1	(a,0,0,0)	*Ama*2	A2	*c*
52	Γ_1	(a)	*P*4*bm*	O1	*d*
53	Γ_1	(a)	*P*4*bm*	O1	*d*
54	Γ_1	(a)	*P*4*bm*	O1	*d*
72	S_1	(a,0,0,0)	*Ama*2	O1	*d*
73	S_1	(a,0,0,0)	*Ama*2	O1	*d*
74	S_1	(a,0,0,0)	*Ama*2	O1	*d*
75	S_1	(a,0,0,0)	*Ama*2	O1	*d*
76	Γ_1	(a)	*P*4*bm*	O2	*d*
77	Γ_1	(a)	*P*4*bm*	O2	*d*
96	S_1	(a,0,0,0)	*Ama*2	O2	*d*
97	S_1	(a,0,0,0)	*Ama*2	O2	*d*
98	S_1	(a,0,0,0)	*Ama*2	O2	*d*
99	S_1	(a,0,0,0)	*Ama*2	O2	*d*
100	Γ_1	(a)	*P*4*bm*	O3	*c*
101	Γ_1	(a)	*P*4*bm*	O3	*c*
112	S_1	(a,0,0,0)	*Ama*2	O3	*c*
114	Γ_1	(a)	*P*4*bm*	O4	*b*
120	S_1	(a,0,0,0)	*Ama*2	O4	*b*
121	S_1	(a,0,0,0)	*Ama*2	O4	*b*
122	Γ_1	(a)	*P*4*bm*	O5	*d*
123	Γ_1	(a)	*P*4*bm*	O5	*d*
124	Γ_1	(a)	*P*4*bm*	O5	*d*
129	M_5	(a,a)	*Pma*2	O5	*d*
135	S_1	(a,0,0,0)	*Ama*2	O5	*d*
136	S_1	(a,0,0,0)	*Ama*2	O5	*d*
137	S_1	(a,0,0,0)	*Ama*2	O5	*d*
138	S_1	(a,0,0,0)	*Ama*2	O5	*d*
139	S_1	(a,0,0,0)	*Ama*2	O5	*d*
140	S_1	(a,0,0,0)	*Ama*2	O5	*d*
141	S_1	(a,0,0,0)	*Ama*2	O5	*d*

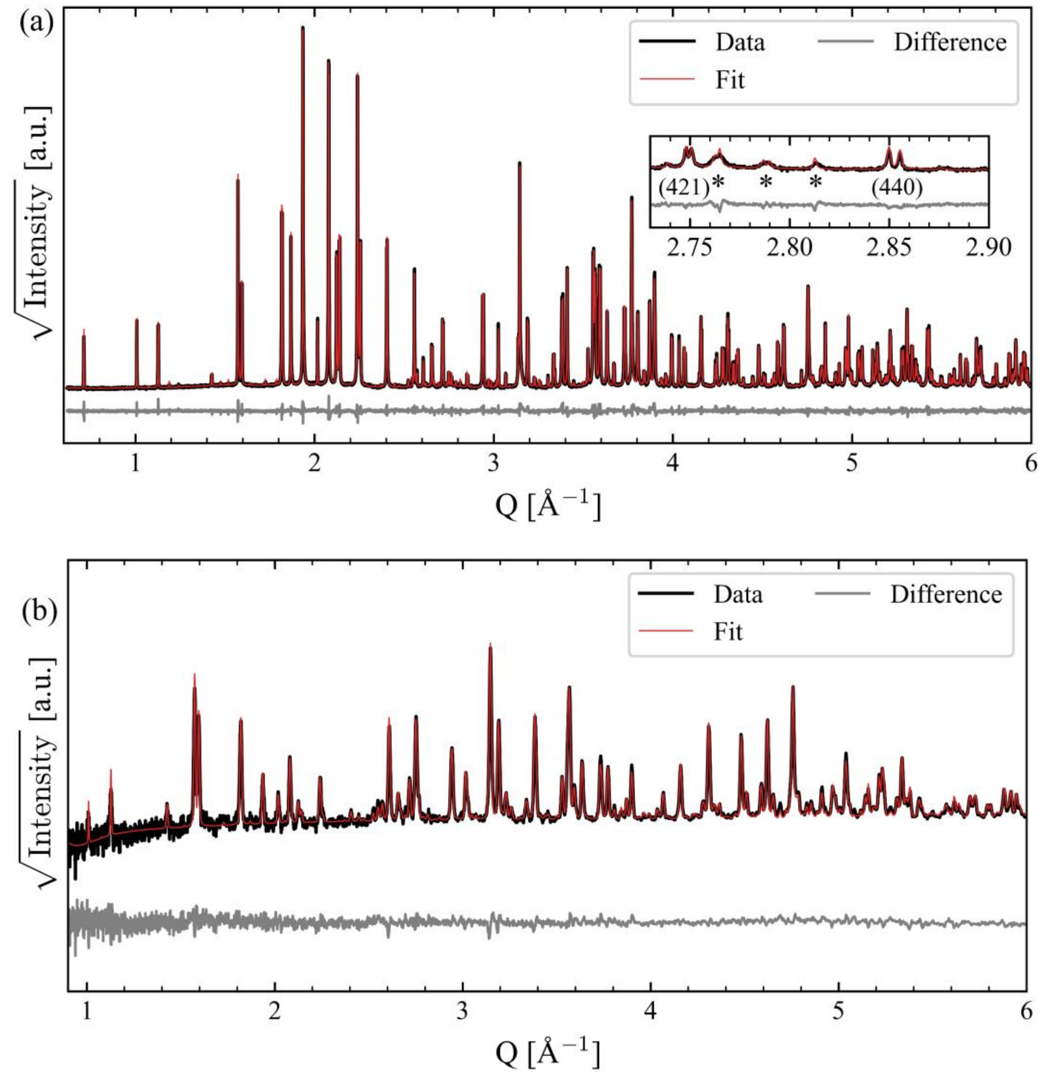

Figure S8. Combined (a) X-ray and (b) neutron Rietveld refinement of room temperature data of BNN using the *Ama*2 space group and the 30 selected symmetry-modes. The insert in panel (a) shows the pseudo-tetragonal (421) and (440) reflections, with the asterisks indicating some reflections characteristic for *Ama*2 relative to the other space groups investigated. R_{wp} = 5.89 %, R_{exp} = 2.04 %, *GOF* = 2.89.

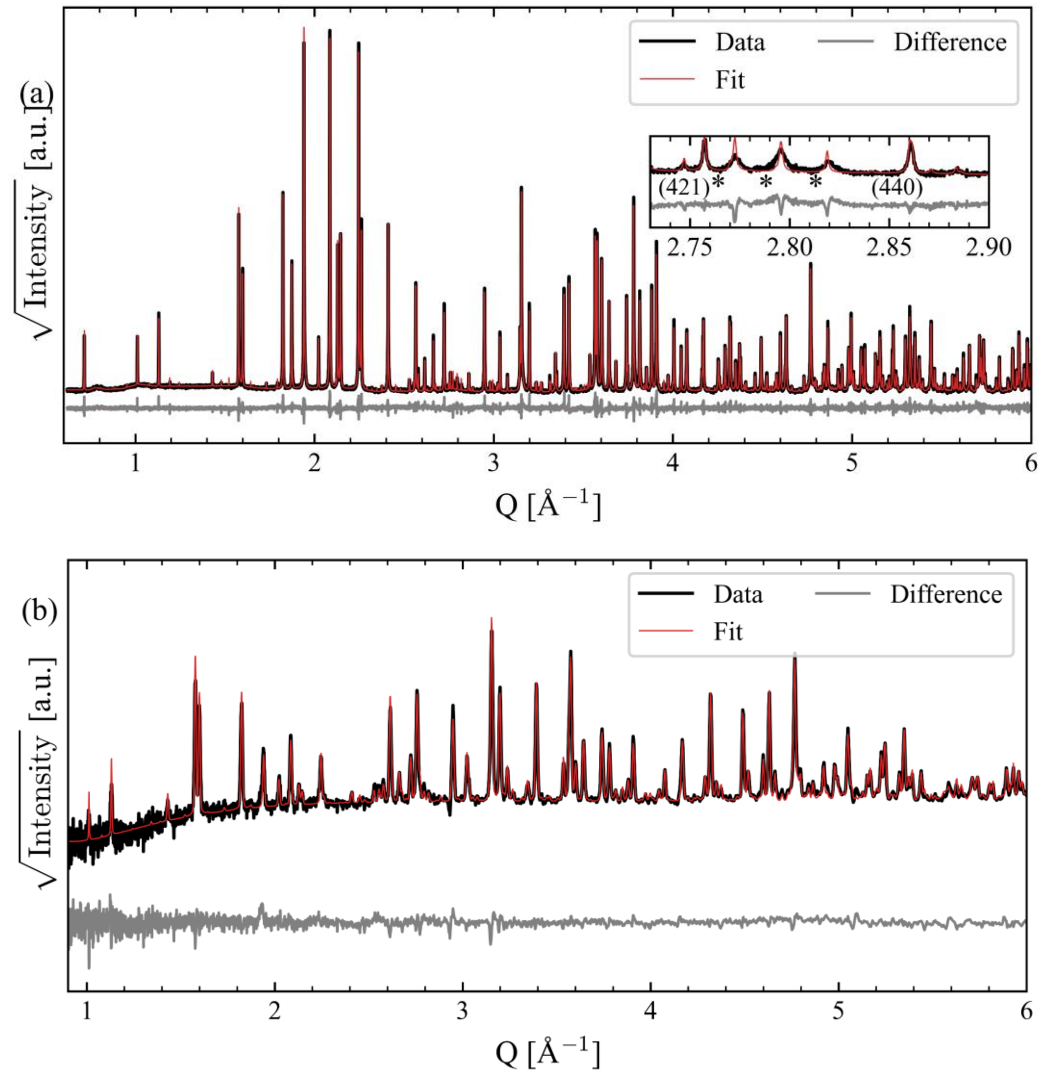

Figure S9. Combined (a) X-ray and (b) neutron Rietveld refinement of the 25 K data of BNN using the *Ama*2 space group and the 24 selected symmetry-modes. Insert in panel (a) shows the pseudo-tetragonal (421) and (440) reflections, with the asterisks indicating some reflections characteristic for *Ama*2 relative to the other space groups investigated. R_{wp} = 9.13 %, R_{exp} = 3.19 %, *GOF* = 2.86.

Pawley fits of selected space groups

Pawley refinements were performed on the room temperature X-ray powder diffraction data of BNN with the following space groups: *Ama*2, *Cmm*2, *Ima*2, $Cmc2_1$ and *I*4; and are presented in Figures S10-S14 respectively. Refined lattice parameters and *R*-values are listed in Table S2. All the space groups, with the exception of *I*4 describes the main features of the diffraction pattern. This can be seen visually in the figures, but also on the small differences in R_{wp} and *GOF* values between the space groups. However, only *Ama*2 also described well the weak features in the diffraction patterns, see inset in Figures S10-S14. Based on this, it is evident that *Ama*2 is the correct space group for the room temperature structure of BNN.

A quick note on the R_{wp} and *GOF* values for the Pawley and Rietveld refinement using the *Ama*2 space group to the very observant readers: these values are higher for the *Ama*2 Pawley fits than the *Ama*2 Rietveld refinements because the asymmetric peak shape was not accounted for when performing the Pawley fits.

Table S2. Space group, lattice parameters, R_{exp}, R_{wp} and *GOF* values from Pawley refinement of room temperature data of BNN using the five different space groups.

Space group	*a* [Å]	*b* [Å]	*c* [Å]	R_{exp} [%]	R_{wp} [%]	*GOF*
*Ama*2	17.638	35.208	7.991	2.24	7.93	3.54
*Cmm*2	17.638	17.604	3.996	2.31	8.48	3.67
*Ima*2	17.638	17.604	7.991	2.29	8.23	3.60
$Cmc2_1$	17.638	17.604	7.991	2.29	8.48	3.70
*I*4	17.636	17.600	7.990	2.29	28.26	12.34

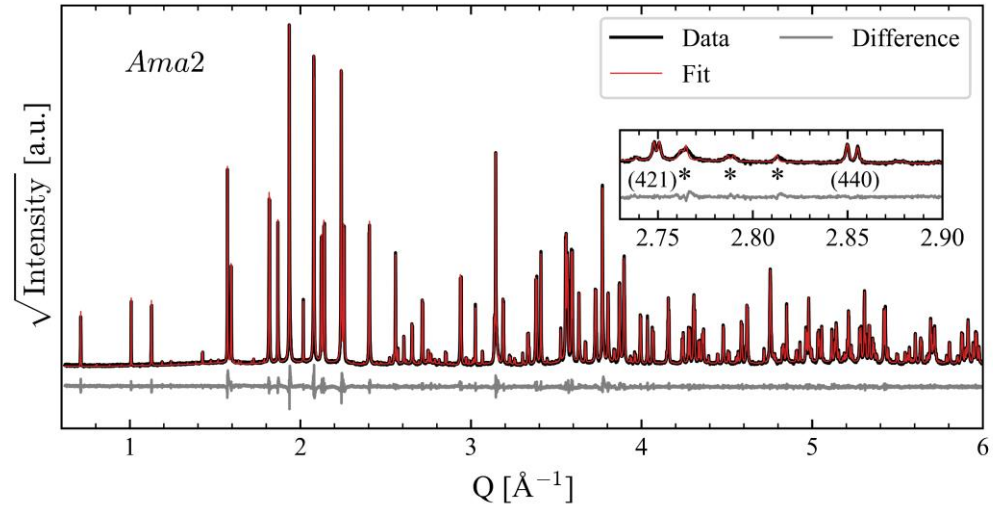

Figure S10. Pawley refinement of the room temperature X-ray powder diffraction data of BNN using the *Ama*2 space group. The insert shows the pseudo-tetragonal (421) and (440) reflections, with the asterisks indicating some reflections characteristic for *Ama*2 relative to the other space groups investigated. R_{wp} = 7.93 %, R_{exp} = 2.24 %, *GOF* = 3.54.

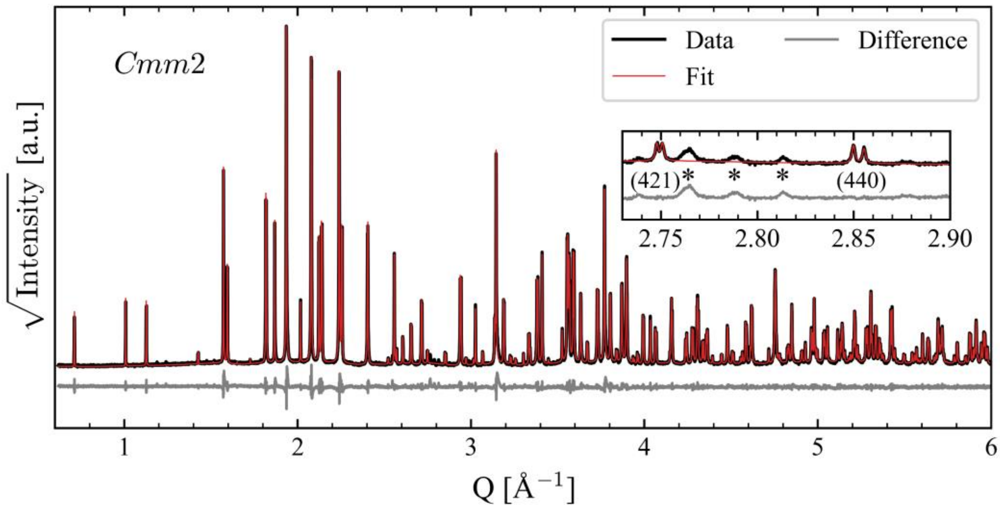

Figure S11. Pawley refinement of the room temperature X-ray powder diffraction data of BNN using the *Cmm*2 space group. The insert shows the pseudo-tetragonal (421) and (440) reflections, with the asterisks indicating some reflections characteristic for *Ama*2 relative to the other space groups investigated. R_{wp} = 8.48 %, R_{exp} = 2.31 %, *GOF* = 3.67.

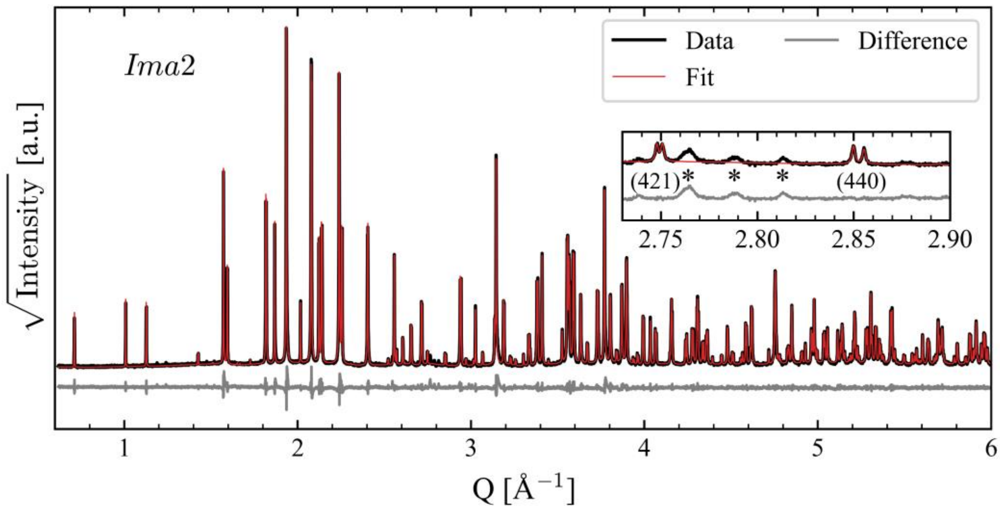

Figure S12. Pawley refinement of the room temperature X-ray powder diffraction data of BNN using the *Ima*2 space group. The insert shows the pseudo-tetragonal (421) and (440) reflections, with the asterisks indicating some reflections characteristic for *Ama*2 relative to the other space groups investigated. R_{wp} = 8.23 %, R_{exp} = 2.29 %, *GOF* = 3.60.

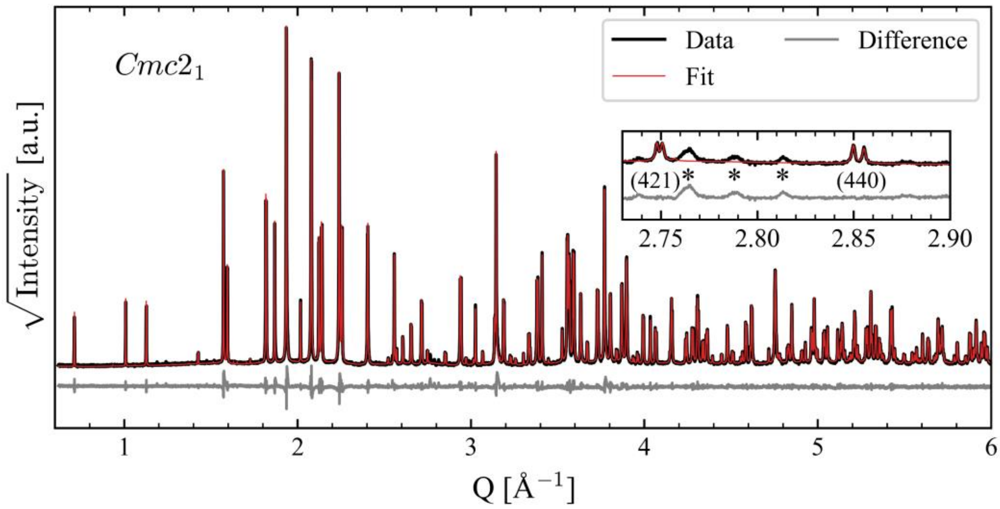

Figure S13. Pawley refinement of the room temperature X-ray powder diffraction data of BNN using the *Cmc*2_1 space group. Insert shows the pseudo-tetragonal (421) and (440) reflections, with the asterisks indicating some reflections characteristic for *Ama*2 relative to the other space groups investigated. R_{wp} = 8.48 %, R_{exp} = 2.29 %, *GOF* = 3.70.

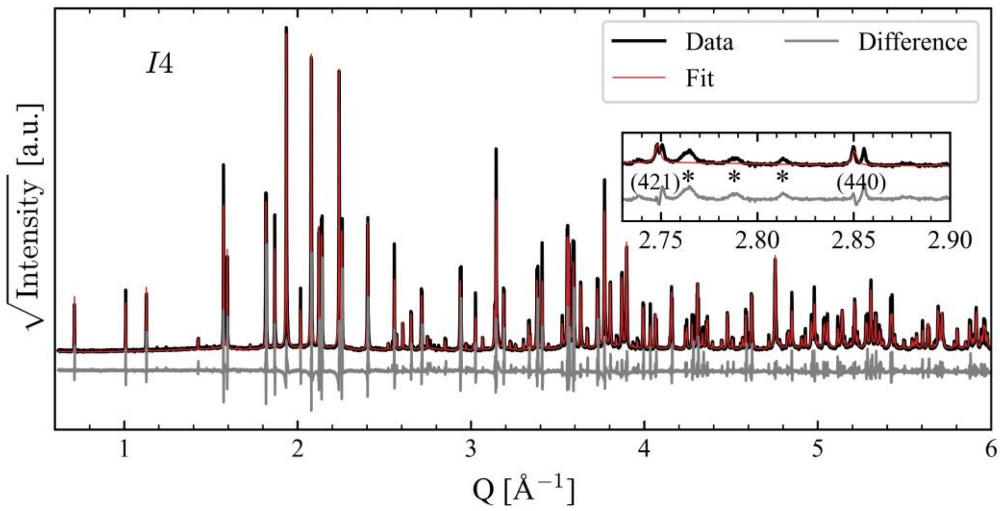

Figure S14. Pawley refinement of the room temperature X-ray powder diffraction data of BNN using the *I*4 space group. Insert shows the pseudo-tetragonal (421) and (440) reflections, with the asterisks indicating some reflections characteristic for *Ama*2 relative to the other space groups investigated. R_{wp} = 28.26 %, R_{exp} = 2.29 %, *GOF* = 12.34.

Refined parameters for BNN with the *Ama*2 space group at room temperature

Table S3. Refined structural parameters for room temperature X-ray powder diffraction data on BNN with the *Ama*2 space group and refining all the allowed 145 atomic *xyz* coordinates. Lattice parameters were a = 17.63794(2), b = 35.20819(4) and c = 7.991382(8), and R-values R_{wp} = 5.94 %, R_{exp} = 2.64 % and GOF=2.25.

site	Wyckoff pos.	atom	*x*	*y*	*z*	Occ.	B_{iso} [Å^2]
Nb1_1	4*b*	Nb	0.75	0.1249(3)	0.0	1	0.429(9)
Nb1_2	4*b*	Nb	0.25	0.3754(3)	0.0	1	0.429(9)
Nb1_3	4*b*	Nb	0.75	0.8750(3)	0.0	1	0.429(9)
Nb1_4	4*b*	Nb	0.25	0.6248(3)	0.0	1	0.429(9)
Nb2_1	8*c*	Nb	0.6424(4)	0.7839(2)	0.0017(7)	1	0.428(5)
Nb2_2	8*c*	Nb	0.3576(4)	0.7155(2)	0.0048(6)	1	0.428(5)
Nb2_3	8*c*	Nb	0.8581(4)	0.9659(2)	-0.0028(7)	1	0.428(5)
Nb2_4	8*c*	Nb	0.1414(4)	0.5341(2)	0.0007(7)	1	0.428(5)
Nb2_5	8*c*	Nb	0.4328(4)	0.8206(2)	-0.0065(7)	1	0.428(5)
Nb2_6	8*c*	Nb	0.5690(4)	0.6786(2)	-0.0038(7)	1	0.428(5)
Nb2_7	8*c*	Nb	0.0674(4)	0.9287(2)	-0.0047(7)	1	0.428(5)
Nb2_8	8*c*	Nb	0.9314(4)	0.5701(2)	-0.0034(7)	1	0.428(5)
A1_1	8*c*	Ba	0.5015(7)	0.7523(3)	0.259(2)	0.0745(9)	1.03(6)
		Na	0.5015(7)	0.7523(3)	0.259(2)	0.9255(9)	1.03(6)
A1_2	4*a*	Ba	0.0	0.0	0.249(4)	0.0745(9)	1.03(6)
		Na	0.0	0.0	0.249(4)	0.9255(9)	1.03(6)
A1_3	4*a*	Ba	0.0	0.5	0.251(4)	0.0745(9)	1.03(6)
		Na	0.0	0.5	0.251(4)	0.9255(9)	1.03(6)
A2_1	8*c*	Ba	0.9215(1)	0.87031(7)	0.2436(8)	0.9628(5)	1.219(9)
		Na	0.9215(1)	0.87031(7)	0.2436(8)	0.0372(5)	1.219(9)

A2_2	8*c*	Ba	0.0776(1)	0.6226(1)	0.2438(8)	0.9628(5)	1.219(9)
		Na	0.0776(1)	0.6226(1)	0.2438(8)	0.0372(5)	1.219(9)
A2_3	4*b*	Ba	0.25	0.9610(1)	0.243(1)	0.9628(5)	1.219(9)
		Na	0.25	0.9610(1)	0.243(1)	0.0372(5)	1.219(9)
A2_4	4*b*	Ba	0.75	0.5386(1)	0.242(1)	0.9628(5)	1.219(9)
		Na	0.75	0.5386(1)	0.242(1)	0.0372(5)	1.219(9)
A2_5	4*b*	Ba	0.25	0.7885(1)	0.243(1)	0.9628(5)	1.219(9)
		Na	0.25	0.7885(1)	0.243(1)	0.0372(5)	1.219(9)
A2_6	4*b*	Ba	0.75	0.7109(1)	0.243(1)	0.9628(5)	1.219(9)
		Na	0.75	0.7109(1)	0.243(1)	0.0372(5)	1.219(9)
O1_1	8*c*	O	0.034(2)	0.876(1)	-0.026(3)	1	0.26(5)
O1_2	8*c*	O	0.966(2)	0.6219(8)	-0.043(2)	1	0.26(5)
O1_3	4*b*	O	0.25	0.0228(9)	-0.023(5)	1	0.26(5)
O1_4	4*b*	O	0.75	0.483(1)	-0.023(5)	1	0.26(5)
O1_5	4*b*	O	0.25	0.234(1)	-0.023(6)	1	0.26(5)
O1_6	4*b*	O	0.75	0.262(1)	-0.028(5)	1	0.26(5)
O2_1	8*c*	O	0.606(2)	0.2353(8)	-0.040(3)	1	0.26(5)
O2_2	8*c*	O	0.396(2)	0.2717(6)	-0.021(4)	1	0.26(5)
O2_3	8*c*	O	0.889(1)	0.0204(6)	-0.069(3)	1	0.26(5)
O2_4	8*c*	O	0.101(2)	0.4841(8)	-0.023(4)	1	0.26(5)
O2_5	8*c*	O	0.536(2)	0.8016(9)	-0.042(3)	1	0.26(5)
O2_6	8*c*	O	0.463(2)	0.6963(9)	-0.015(4)	1	0.26(5)
O2_7	8*c*	O	0.971(1)	0.9508(6)	-0.002(4)	1	0.26(5)
O2_8	8*c*	O	0.040(1)	0.5582(6)	-0.047(4)	1	0.26(5)

O3_1	8*c*	O	0.677(2)	0.1683(8)	-0.020(4)	1	0.26(5)
O3_2	8*c*	O	0.323(2)	0.334(1)	-0.042(4)	1	0.26(5)
O3_3	8*c*	O	0.824(2)	0.0834(9)	-0.045(5)	1	0.26(5)
O3_4	8*c*	O	0.173(1)	0.4117(6)	-0.018(4)	1	0.26(5)
O3_5	8*c*	O	0.667(2)	0.8381(7)	-0.059(3)	1	0.26(5)
O3_6	8*c*	O	0.328(2)	0.6617(8)	-0.001(4)	1	0.26(5)
O3_7	8*c*	O	0.837(1)	0.9119(8)	-0.008(4)	1	0.26(5)
O3_8	8*c*	O	0.170(2)	0.5858(9)	-0.034(3)	1	0.26(5)
O4_1	8*c*	O	0.869(1)	0.7914(4)	0.230(5)	1	0.26(5)
O4_2	8*c*	O	0.1387(9)	0.7280(4)	0.240(6)	1	0.26(5)
O4_3	8*c*	O	0.6509(9)	0.9707(4)	0.226(4)	1	0.26(5)
O4_4	8*c*	O	0.363(1)	0.5378(5)	0.216(4)	1	0.26(5)
O4_5	8*c*	O	0.422(1)	0.9387(3)	0.229(5)	1	0.26(5)
O4_6	8*c*	O	0.5472(8)	0.5704(5)	0.221(4)	1	0.26(5)
O4_7	8*c*	O	0.078(1)	0.8211(4)	0.223(4)	1	0.26(5)
O4_8	8*c*	O	0.936(1)	0.6767(4)	0.227(5)	1	0.26(5)
O5_1	4*b*	O	0.75	0.8698(6)	0.259(5)	1	0.26(5)
O5_2	4*b*	O	0.25	0.6127(5)	0.281(4)	1	0.26(5)
O5_3	4*b*	O	0.75	0.1149(6)	0.217(4)	1	0.26(5)
O5_4	4*b*	O	0.25	0.3701(7)	0.248(7)	1	0.26(5)

Temperature dependent symmetry mode amplitudes

The temperature dependence of the 40 modes in the final mode set obtained from the batch sequential Rietveld refinement is shown in Figures S15-17.

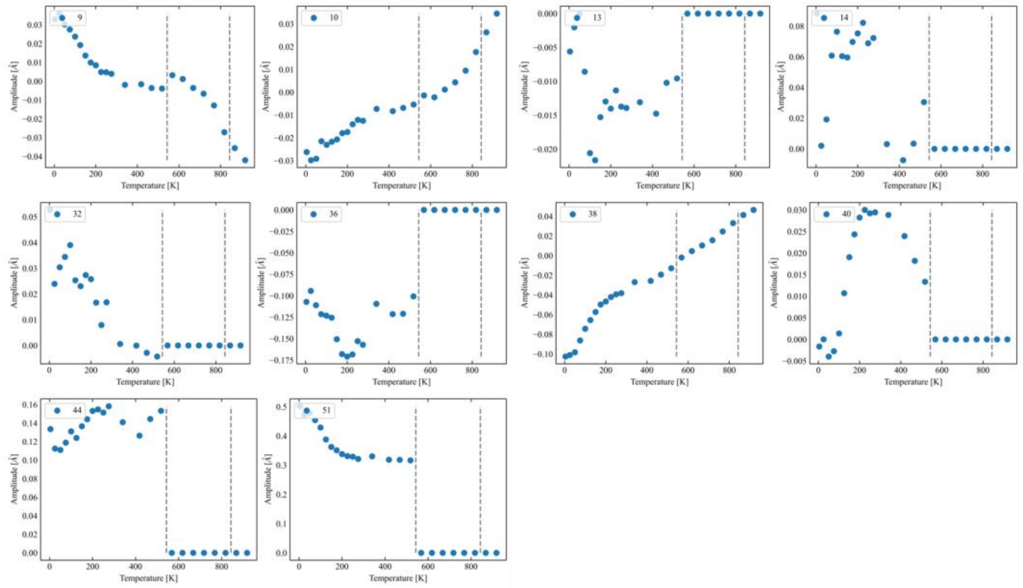

Figure S15. Temperature evolution of the modes in the final mode set acting on the cations in BNN. With respect to the polar *P4bm* structure of BNN, modes 9, 10, 13, 14 and 32 acts on B2/d, mode 36 on A1/a and 38, 40, 44 and 51 on A2/c.

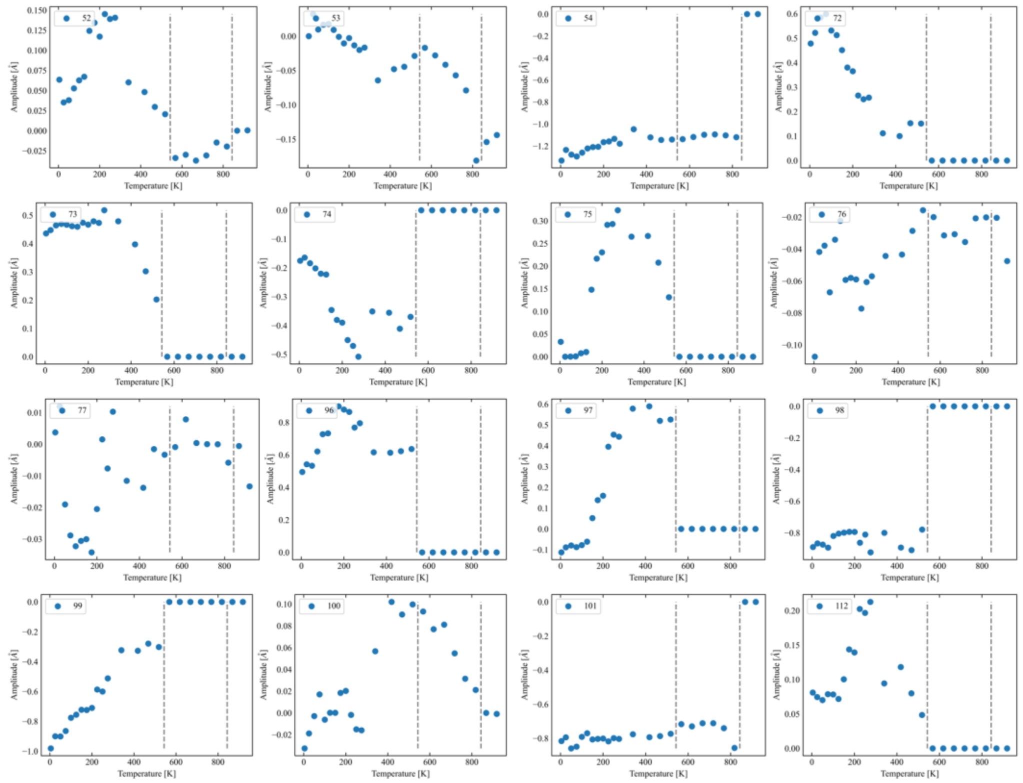

Figure S16. Temperature evolution of the modes in the final mode set acting on the O1, O2, and O3 oxygen sites in BNN. With respect to the polar *P4bm* structure of BNN, modes 52, 53, 54, 72, 73, 74 and 75 acts on O1/d, modes 76, 77, 96, 97, 98 and 99 on O2/d and modes 100, 101 and 112 acts on O3/c.

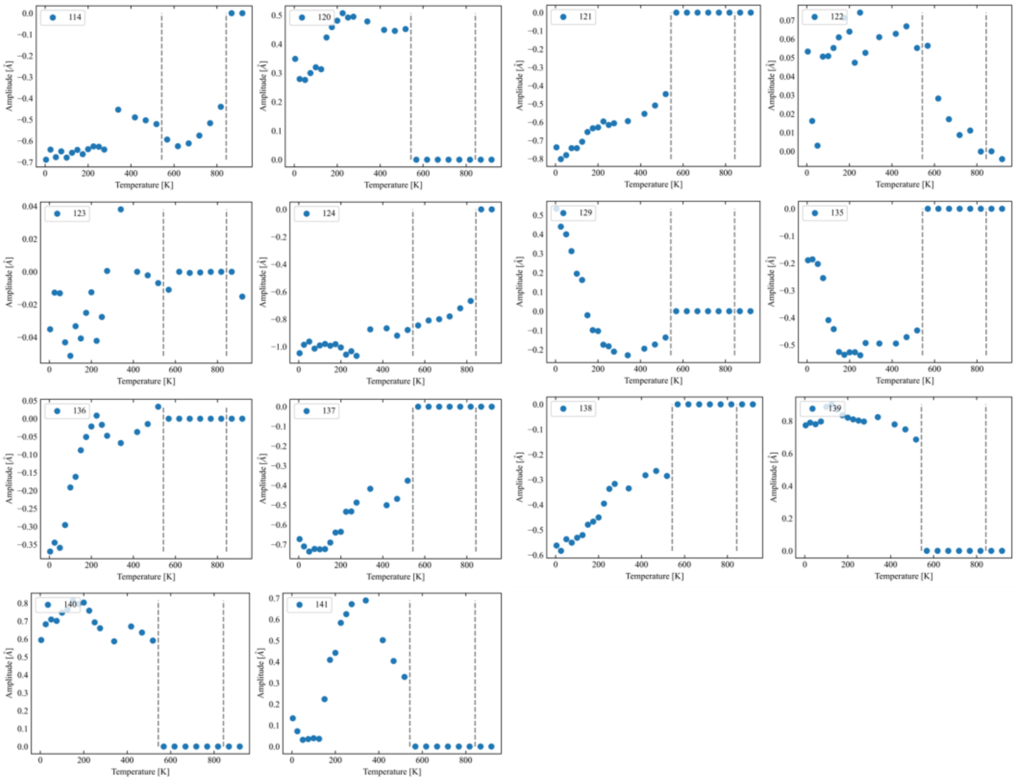

Figure S17. Temperature evolution of the modes in the final mode set acting on the O4 and O5 oxygen sites in BNN. With respect to the polar *P*4*bm* structure of BNN, modes 114, 120 and 121 acts on O4/b, modes 122, 123, 124, 129, 135, 136, 137, 138, 139, 140 and 141 on O5/d.

Pawley fit of an incommensurate structure

As mentioned in the introduction of the main text, BNN is known to show satellite reflections below the ferroelastic transition with single crystal diffraction. This is a common feature for tetragonal tungsten bronzes, for example the incommensurately modulated orthorhombic structure of $Ca_{0.24}Ba_{0.76}Nb_2O_6$ (CBN24) [6] and $Sr_{0.82}Ba_{0.18}Nb_2O_6$ (SBN82) [7] are reported to be *A*2*mm*(½0γ)000 (super-space group no. 35.1.16.9) with modulation vectors of **q** = 0.6638**c*** and **q** = 0.2931**c*** respectively. However, the incommensurate structure(s) of BNN has not been solved to the authors' knowledge.

In this work we used *Jana2020* [8] to perform a Pawley fit with the *A*2*mm*(½0γ)000 space group, refining the lattice parameters, **q**-vector, a scale factor, zero shift and 4 Pseudo-Voigt peak shape parameters. The refinement was performed with 0th, 1st and 2nd order satellite reflections, the results of which are shown in Figure S18. The 0th order refinement shows no improvement over the *Cmm2* space group as expected, while especially when including 2nd order satellite reflections some of the "characteristic" *Ama2* reflections are fitted. Based on this, and the fact that measuring 2nd order satellite reflections with X-ray powder diffraction, we concluded that our data are better described with the average *Ama2* space group than the incommensurately modulated superstructure.

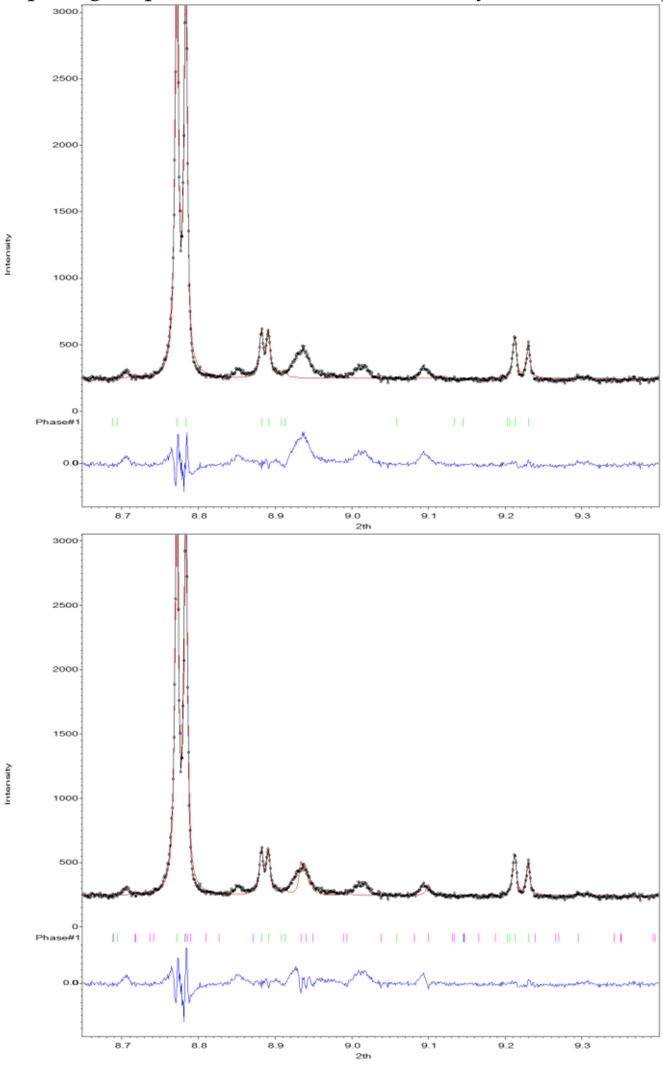

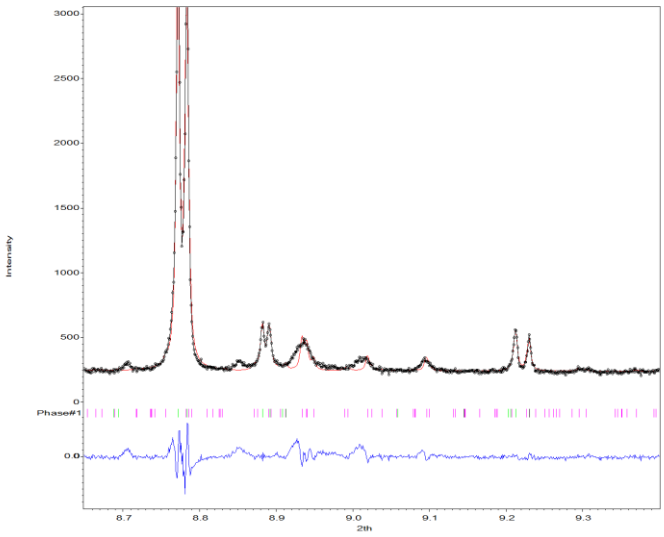

Figure S18. Room temperature X-ray powder diffraction data of BNN fitted with a Pawley fit using the super-space group *A*2*mm*(½0γ)000, allowing for 0th (top), 1st (middle) and 2nd (bottom) order satellite reflections. R_{wp} values were 7.96, 7.24 and 6.65 % respectively.

Full list of symmetry modes for *Ama*2

Table S4. Overview of all the 145 symmetry modes for BNN in the *Ama*2 space group, including the mode number, the k-vector components, the irreducible representation (IRREP), the components of the order parameter direction vector of the IRREP, the site and Wyckoff position the mode is acting on, and the IRREP point group.

Mode #	k-vector components	IRREP	IRREP direction	Atom:Wyckoff pos.	IRREP point group
1	[0,0,0]	Γ_1	(a)	Nb1:b	A1
2	[0,0,0]	Γ_3	(a)	Nb1:b	A1
3	[1/2,1/2,0]	M_5	(a,a)	Nb1:b	B2
4	[1/2,1/2,0]	M_5	(a,a)	Nb1:b	B1
5	[1/4,1/4,1/2]	S_1	(a,0;0,0)	Nb1:b	A1_1
6	[1/4,1/4,1/2]	S_1	(a,0;0,0)	Nb1:b	A1_2
7	[1/4,1/4,1/2]	S_1	(a,0;0,0)	Nb1:b	B2
8	[1/4,1/4,1/2]	S_1	(a,0;0,0)	Nb1:b	B1
9	[0,0,0]	Γ_1	(a)	Nb2:d	A_1
10	[0,0,0]	Γ_1	(a)	Nb2:d	A_2
11	[0,0,0]	Γ_1	(a)	Nb2:d	A_3
12	[0,0,0]	Γ_3	(a)	Nb2:d	A_1
13	[0,0,0]	Γ_3	(a)	Nb2:d	A_2
14	[0,0,0]	Γ_3	(a)	Nb2:d	A_3
15	[1/2,1/2,0]	M_5	(a,a)	Nb2:d	A_1
16	[1/2,1/2,0]	M_5	(a,a)	Nb2:d	A_2
17	[1/2,1/2,0]	M_5	(a,a)	Nb2:d	A_3
18	[1/2,1/2,0]	M_5	(a,a)	Nb2:d	A_4
19	[1/2,1/2,0]	M_5	(a,a)	Nb2:d	A_5
20	[1/2,1/2,0]	M_5	(a,a)	Nb2:d	A_6
21	[1/4,1/4,1/2]	S_1	(a,0;0,0)	Nb2:d	A_1
22	[1/4,1/4,1/2]	S_1	(a,0;0,0)	Nb2:d	A_2
23	[1/4,1/4,1/2]	S_1	(a,0;0,0)	Nb2:d	A_3
24	[1/4,1/4,1/2]	S_1	(a,0;0,0)	Nb2:d	A_4
25	[1/4,1/4,1/2]	S_1	(a,0;0,0)	Nb2:d	A_5
26	[1/4,1/4,1/2]	S_1	(a,0;0,0)	Nb2:d	A_6
27	[1/4,1/4,1/2]	S_1	(a,0;0,0)	Nb2:d	A_7
28	[1/4,1/4,1/2]	S_1	(a,0;0,0)	Nb2:d	A_8
29	[1/4,1/4,1/2]	S_1	(a,0;0,0)	Nb2:d	A_9
30	[1/4,1/4,1/2]	S_1	(a,0;0,0)	Nb2:d	A_10
31	[1/4,1/4,1/2]	S_1	(a,0;0,0)	Nb2:d	A_11
32	[1/4,1/4,1/2]	S_1	(a,0;0,0)	Nb2:d	A_12
33	[0,0,0]	Γ_1	(a)	A1:a	A
34	[1/2,1/2,0]	M_5	(a,a)	A1:a	A
35	[1/4,1/4,1/2]	S_1	(a,0;0,0)	A1:a	A
36	[1/4,1/4,1/2]	S_1	(a,0;0,0)	A1:a	E*_1

37	[1/4,1/4,1/2]	S1	(a,0;0,0)	A1:a	E*_2
38	[0,0,0]	Γ_1	(a)	A2:c	A'_1
39	[0,0,0]	Γ_1	(a)	A2:c	A'_2
40	[0,0,0]	Γ_3	(a)	A2:c	A'_1
41	[0,0,0]	Γ_3	(a)	A2:c	A'_2
42	[1/2,1/2,0]	M_5	(a,a)	A2:c	A'_1
43	[1/2,1/2,0]	M_5	(a,a)	A2:c	A'_2
44	[1/2,1/2,0]	M_5	(a,a)	A2:c	A''
45	[1/4,1/4,1/2]	S_1	(a,0;0,0)	A2:c	A'_1
46	[1/4,1/4,1/2]	S_1	(a,0;0,0)	A2:c	A'_2
47	[1/4,1/4,1/2]	S_1	(a,0;0,0)	A2:c	A'_3
48	[1/4,1/4,1/2]	S_1	(a,0;0,0)	A2:c	A'_4
49	[1/4,1/4,1/2]	S_1	(a,0;0,0)	A2:c	A'_5
50	[1/4,1/4,1/2]	S_1	(a,0;0,0)	A2:c	A'_6
51	[1/4,1/4,1/2]	S_1	(a,0;0,0)	A2:c	A''
52	[0,0,0]	Γ_1	(a)	O1:d	A_1
53	[0,0,0]	Γ_1	(a)	O1:d	A_2
54	[0,0,0]	Γ_1	(a)	O1:d	A_3
55	[0,0,0]	Γ_3	(a)	O1:d	A_1
56	[0,0,0]	Γ_3	(a)	O1:d	A_2
57	[0,0,0]	Γ_3	(a)	O1:d	A_3
58	[1/2,1/2,0]	M_5	(a,a)	O1:d	A_1
59	[1/2,1/2,0]	M_5	(a,a)	O1:d	A_2
60	[1/2,1/2,0]	M_5	(a,a)	O1:d	A_3
61	[1/2,1/2,0]	M_5	(a,a)	O1:d	A_4
62	[1/2,1/2,0]	M_5	(a,a)	O1:d	A_5
63	[1/2,1/2,0]	M_5	(a,a)	O1:d	A_6
64	[1/4,1/4,1/2]	S_1	(a,0;0,0)	O1:d	A_1
65	[1/4,1/4,1/2]	S_1	(a,0;0,0)	O1:d	A_2
66	[1/4,1/4,1/2]	S_1	(a,0;0,0)	O1:d	A_3
67	[1/4,1/4,1/2]	S_1	(a,0;0,0)	O1:d	A_4
68	[1/4,1/4,1/2]	S_1	(a,0;0,0)	O1:d	A_5
69	[1/4,1/4,1/2]	S_1	(a,0;0,0)	O1:d	A_6
70	[1/4,1/4,1/2]	S_1	(a,0;0,0)	O1:d	A_7
71	[1/4,1/4,1/2]	S_1	(a,0;0,0)	O1:d	A_8
72	[1/4,1/4,1/2]	S_1	(a,0;0,0)	O1:d	A_9
73	[1/4,1/4,1/2]	S_1	(a,0;0,0)	O1:d	A_10
74	[1/4,1/4,1/2]	S_1	(a,0;0,0)	O1:d	A_11
75	[1/4,1/4,1/2]	S_1	(a,0;0,0)	O1:d	A_12
76	[0,0,0]	Γ_1	(a)	O2:d	A_1
77	[0,0,0]	Γ_1	(a)	O2:d	A_2
78	[0,0,0]	Γ_1	(a)	O2:d	A_3
79	[0,0,0]	Γ_3	(a)	O2:d	A_1

80	[0,0,0]	Γ_3	(a)	O2:d	A_2
81	[0,0,0]	Γ_3	(a)	O2:d	A_3
82	[1/2,1/2,0]	M_5	(a,a)	O2:d	A_1
83	[1/2,1/2,0]	M_5	(a,a)	O2:d	A_2
84	[1/2,1/2,0]	M_5	(a,a)	O2:d	A_3
85	[1/2,1/2,0]	M_5	(a,a)	O2:d	A_4
86	[1/2,1/2,0]	M_5	(a,a)	O2:d	A_5
87	[1/2,1/2,0]	M_5	(a,a)	O2:d	A_6
88	[1/4,1/4,1/2]	S_1	(a,0;0,0)	O2:d	A_1
89	[1/4,1/4,1/2]	S_1	(a,0;0,0)	O2:d	A_2
90	[1/4,1/4,1/2]	S_1	(a,0;0,0)	O2:d	A_3
91	[1/4,1/4,1/2]	S_1	(a,0;0,0)	O2:d	A_4
92	[1/4,1/4,1/2]	S_1	(a,0;0,0)	O2:d	A_5
93	[1/4,1/4,1/2]	S_1	(a,0;0,0)	O2:d	A_6
94	[1/4,1/4,1/2]	S_1	(a,0;0,0)	O2:d	A_7
95	[1/4,1/4,1/2]	S_1	(a,0;0,0)	O2:d	A_8
96	[1/4,1/4,1/2]	S_1	(a,0;0,0)	O2:d	A_9
97	[1/4,1/4,1/2]	S_1	(a,0;0,0)	O2:d	A_10
98	[1/4,1/4,1/2]	S_1	(a,0;0,0)	O2:d	A_11
99	[1/4,1/4,1/2]	S_1	(a,0;0,0)	O2:d	A_12
100	[0,0,0]	Γ_1	(a)	O3:c	A'_1
101	[0,0,0]	Γ_1	(a)	O3:c	A'_2
102	[0,0,0]	Γ_3	(a)	O3:c	A'_1
103	[0,0,0]	Γ_3	(a)	O3:c	A'_2
104	[1/2,1/2,0]	M_5	(a,a)	O3:c	A'_1
105	[1/2,1/2,0]	M_5	(a,a)	O3:c	A'_2
106	[1/2,1/2,0]	M_5	(a,a)	O3:c	A''
107	[1/4,1/4,1/2]	S_1	(a,0;0,0)	O3:c	A'_1
108	[1/4,1/4,1/2]	S_1	(a,0;0,0)	O3:c	A'_2
109	[1/4,1/4,1/2]	S_1	(a,0;0,0)	O3:c	A'_3
110	[1/4,1/4,1/2]	S_1	(a,0;0,0)	O3:c	A'_4
111	[1/4,1/4,1/2]	S_1	(a,0;0,0)	O3:c	A'_5
112	[1/4,1/4,1/2]	S_1	(a,0;0,0)	O3:c	A'_6
113	[1/4,1/4,1/2]	S_1	(a,0;0,0)	O3:c	A''
114	[0,0,0]	Γ_1	(a)	O4:b	A1
115	[0,0,0]	Γ_1	(a)	O4:b	A1
116	[1/2,1/2,0]	M_5	(a,a)	O4:b	B2
117	[1/2,1/2,0]	M_5	(a,a)	O4:b	B1
118	[1/4,1/4,1/2]	S_1	(a,0;0,0)	O4:b	A1_1
119	[1/4,1/4,1/2]	S_1	(a,0;0,0)	O4:b	A1_2
120	[1/4,1/4,1/2]	S_1	(a,0;0,0)	O4:b	B2
121	[1/4,1/4,1/2]	S_1	(a,0;0,0)	O4:b	B1
122	[0,0,0]	Γ_1	(a)	O5:d	A_1

123	[0,0,0]	Γ_1	(a)	O5:d	A_2
124	[0,0,0]	Γ_1	(a)	O5:d	A_3
125	[0,0,0]	Γ_3	(a)	O5:d	A_1
126	[0,0,0]	Γ_3	(a)	O5:d	A_2
127	[0,0,0]	Γ_3	(a)	O5:d	A_3
128	[1/2,1/2,0]	M_5	(a,a)	O5:d	A_1
129	[1/2,1/2,0]	M_5	(a,a)	O5:d	A_2
130	[1/2,1/2,0]	M_5	(a,a)	O5:d	A_3
131	[1/2,1/2,0]	M_5	(a,a)	O5:d	A_4
132	[1/2,1/2,0]	M_5	(a,a)	O5:d	A_5
133	[1/2,1/2,0]	M_5	(a,a)	O5:d	A_6
134	[1/4,1/4,1/2]	S_1	(a,0;0,0)	O5:d	A_1
135	[1/4,1/4,1/2]	S_1	(a,0;0,0)	O5:d	A_2
136	[1/4,1/4,1/2]	S_1	(a,0;0,0)	O5:d	A_3
137	[1/4,1/4,1/2]	S_1	(a,0;0,0)	O5:d	A_4
138	[1/4,1/4,1/2]	S_1	(a,0;0,0)	O5:d	A_5
139	[1/4,1/4,1/2]	S_1	(a,0;0,0)	O5:d	A_6
140	[1/4,1/4,1/2]	S_1	(a,0;0,0)	O5:d	A_7
141	[1/4,1/4,1/2]	S_1	(a,0;0,0)	O5:d	A_8
142	[1/4,1/4,1/2]	S_1	(a,0;0,0)	O5:d	A_9
143	[1/4,1/4,1/2]	S_1	(a,0;0,0)	O5:d	A_10
144	[1/4,1/4,1/2]	S_1	(a,0;0,0)	O5:d	A_11
145	[1/4,1/4,1/2]	S_1	(a,0;0,0)	O5:d	A_12

References

1. Floquet, N. *et al.* Ferroelectric domain walls in $BaTiO_3$: Fingerprints in XRPD diagrams and quantitative HRTEM image analysis. *J. Phys. III* **7**, 1105–1128 (1997).

2. Boysen, H. Ferroelastic phase transitions and domain structures in powders. *Z. Kristallogr.* **220**, 726–734 (2005).

3. Kerman, S. *et al.* The superstructure determination of displacive distortions via symmetry-mode analysis. *Acta Crystallogr. Sect. A Found. Crystallogr.* **68**, 222–234 (2012).

4. Stokes, H. T., Hatch, D. M. & Campbell, B. J. ISODISTORT, ISOTROPY Software Suite. *ISODISTORT, ISOTROPY Softw. Suite, iso.byu.edu*.

5. Campbell, B. J., Stokes, H. T., Tanner, D. E. & Hatch, D. M. ISODISPLACE: A web-based tool for exploring structural distortions. *J. Appl. Crystallogr.* **39**, 607–614 (2006).

6. Graetsch, H. A., Pandey, C. S., Schreuer, J., Burianek, M. & Muhlberg, M. Incommensurate modulations of relaxor ferroelectric $Ca_{0.24}Ba_{0.76}Nb_2O_6$ (CBN24) and $Ca_{0.31}Ba_{0.69}Nb_2O_6$ (CBN31). *Acta Crystallogr. Sect. B* **70**, 743–749 (2014).

7. Graetsch, H. A. Large structural modulations in the relaxor ferroelectric and intermediate state of strontium rich members (x>0.6) of the $Sr_xBa_{1-x}Nb_2O_6$ (sbn) solid solution series. *J. Solid State Chem.* **246**, 167–175 (2017).

8. Petrícek, V., Dušek, M. & Palatinus, L. Crystallographic computing system JANA2006: General features. *Z. Kristallogr. - Cryst. Mater.* **229**, 345–352 (2014).
